# Using the combined analysis of transcripts and metabolites to propose key genes for differential terpene accumulation across two regions

**DOI:** 10.1186/s12870-015-0631-1

**Published:** 2015-10-06

**Authors:** Ya-Qin Wen, Gan-Yuan Zhong, Yuan Gao, Yi-Bin Lan, Chang-Qing Duan, Qiu-Hong Pan

**Affiliations:** Centre for Viticulture and Enology, College of Food Science and Nutritional Engineering, China Agricultural University, Beijing, 100083 China; United States Department of Agriculture-Agricultural Research Service, Grape Genetics Research Unit, Geneva, NY 14456 USA; Bee Product Quality Supervision and Testing Center, Bee Research Institute, Chinese Academy of Agricultural Sciences, Beijing, 100093 China

**Keywords:** Terpene profiling, Transcriptome, Monoterpenol glucosyltransferases, Aromatic grape variety

## Abstract

**Background:**

Terpenes are of great interest to winemakers because of their extremely low perception thresholds and pleasant floral odors. Even for the same variety, terpene profile can be substantially different for grapevine growing environments. Recently a series of genes required for terpene biosynthesis were biochemically characterized in grape berries. However, the genes that dominate the differential terpene accumulation of grape berries between regions have yet to be identified.

**Methods:**

Free and glycosidically-bound terpenes were identified and quantified using gas chromatography-mass spectrometry (GC-MS) technique. The transcription expression profiling of the genes was obtained by RNA sequencing and part of the results were verified by quantitative real time PCR (QPCR). The gene co-expression networks were constructed with the Cytoscape software v 2.8.2 (www.cytoscape.org).

**Results:**

‘Muscat Blanc a Petits Grains’ berries were collected from two wine-producing regions with strikingly different climates, Gaotai (GT) in Gansu Province and Changli (CL) in Hebei Province in China, at four developmental stages for two consecutive years. GC-MS analysis demonstrated that both free and glycosidically bound terpenes accumulated primarily after veraison and that mature grape berries from CL contained significantly higher concentrations of free and glycosidically bound terpenes than berries from GT. Transcriptome analysis revealed that some key genes involved in terpene biosynthesis were markedly up-regulated in the CL region. Particularly in the MEP pathway, the expression of *VviHDR* (1-hydroxy-2-methyl-2-butenyl 4-diphosphate reductase) paralleled with the accumulation of terpenes, which can promote the flow of isopentenyl diphosphate (IPP) into the terpene synthetic pathway. The glycosidically bound monoterpenes accumulated differentially along with maturation in both regions, which is synchronous with the expression of a monoterpene glucosyltransferase gene (*VviUGT85A2L4* (*VviGT14*)). Other genes were also found to be related to the differential accumulation of terpenes and monoterpene glycosides in the grapes between regions. Transcription factors that could regulate terpene synthesis were predicted through gene co-expression network analysis. Additionally, the genes involved in abscisic acid (ABA) and ethylene signal responses were expressed at high levels earlier in GT grapes than in CL grapes.

**Conclusions:**

Differential production of free and glycosidically-bound terpenes in grape berries across GT and CL regions should be related at least to the expression of both *VviHDR* and *VviUGT85A2L4* (*VviGT14*). Considering the expression patterns of both transcription factors and mature-related genes, we infer that less rainfall and stronger sunshine in the GT region could initiate the earlier expression of ripening-related genes and accelerate the berry maturation, eventually limiting the production of terpene volatiles.

**Electronic supplementary material:**

The online version of this article (doi:10.1186/s12870-015-0631-1) contains supplementary material, which is available to authorized users.

## Background

Terpene volatiles in grape berries are major contributors to the floral/fruity odors of wine and are responsible for the varietal flavor of aromatic wines [1, 2]. Terpenes in grapes are present in both free and glycosidically bound forms. In general, the glycosidically bound form exists much more abundant than the free form [3, 4]. Free-form terpenes directly contribute to aroma odor, whereas non-volatile and flavorless bound-form terpenes are potential contributors to wine aroma odors because they can be converted into free volatile compounds through acidic and enzymatic hydrolysis during wine making [5, 6]. The profiles of volatiles in muscat-type grape varieties have been widely studied [7–10], which indicates that most terpene compounds accumulate as grapes ripen [11]. The typical muscat-like aromas are primarily attributed to a large amount of C10 terpenoids (monoterpenes). The concentrations of terpene volatiles in a berry are affected by many factors, such as grape variety, maturity degree, vintage and vineyard management techniques [12–17]. The same variety, when grown in different climates and regions, can have different aromatic profiles [18, 19], which results in a great difference in the aromatic quality of the wines produced [18, 20]. However, limited attention has been paid to regional variation in terpene compounds in grapes; how and by what mechanism the climate or regional factors affect the expression of related genes and the production of terpenes have not been elucidated yet.

The terpene biosynthetic pathway and the genes involved are generally well known. Terpenes are derived from two common inter-convertible five-carbon (C5) precursors: isopentenyl diphosphate (IPP) and its isomer dimethylallyl diphosphate (DMAPP) [21]. In plants, these C5 precursors are synthesized from two independent pathways: the plastidial 2-methyl-D-erythritol-4-phosphate phosphate (MEP) and the cytoplasmic mevalonic acid (MVA) pathways [22, 23]. The MEP pathway offers substrates for the synthesis of monoterpenes and diterpenes, whereas the MVA pathway provides metabolic precursors for the synthesis of sesquiterpenes (C15) [24, 25]. Recently, an isotope labeling experiment demonstrated that a cross-flow of metabolites exists between the MVA and MEP pathways in some plants [26]. IPP and short prenyl diphosphates might connect the MVA and MEP pathways of isoprenoid metabolism upstream [27]. Among the isoprenoid metabolites, monoterpenes are the greatest contributors to the aromas of white wines made from Muscat and aromatic non-Muscat varieties [28, 29]. Herein, our main concern regards the production of monoterpenes in grapes.

1-Deoxy-D-xylulose 5-phosphate synthase (DXS) is an entrance enzyme to the MEP pathway, catalyzing the condensation of glyceraldehyde-3-phosphate and pyruvate into 1-deoxy-D-xylulose 5-phosphate (DXP). DXP is further converted into geranyl pyrophosphate (GPP, C10) through six enzymatic reactions. At least three rate-limiting enzymes exist in the MEP pathway, including DXS, DXP reducto-isomerase (DXR), and1-hydroxy-2-methyl-2-butenyl 4-diphosphate (HMBPP) reductase (HDR) [30–32]. DXS is a key rate-limiting enzyme in several plant species [31]. The over-expression of *DXS* results in an obvious increase in isoprenoid end products in *Arabidopsis* [33]. Additionally, the accumulation of *VviDXS* transcripts is positively correlated with the concentration of monoterpenes in grapes [34, 35]. Quantitative trait loci (QTL) analysis revealed that the expression of *VviDXS* strongly correlates with the muscat-flavor intensity of grape berries [36]. Also, the expression of *VviHDR* was associated with the accumulation of monoterpenols at the veraison stage of grape berries [11].

As the final enzymes of the terpene biosynthetic pathway, terpene synthases (TPSs) are a large gene family that is responsible for the production of hemiterpenes (C5), monoterpenes (C10), sesquiterpenes (C15) or diterpenes (C20) from the substrates DMAPP, GPP, FPP or GGPP, respectively [37]. Primary monoterpene skeletons can be further modified by the actions of other classes of enzymes, such as cytochrome P450 hydroxylases, dehydrogenases (alcohol and aldehyde oxido-reductases), reductases, glycosyl-transferases and methyl-transferases [38]. The analysis of the *V. vinifera* 12-fold coverage genome sequence predicted 69 putatively functional *VviTPS*s [39]. To date, 43 full-length *VviTPSs* have been biochemically characterized, and their reaction products cover most of the monoterpene and sesquiterpene volatiles in grape berries [39–41]. In aromatic ‘Gewürztraminer’ grapes, an increase in gene transcripts of the terpene biosynthetic pathway upstream correlated with the onset of monoterpenol glycoside accumulation [11]. In other two aromatic grape varieties (Moscato Bianco and Aleatico Aromatic), the highest expression of *VviTPS* genes belonging to the *TPS*-*a* and *TPS*-*b* subfamilies also well corresponded to the peak of free terpene concentrations. In the TPS-g subfamily, only *VviPNLinNer1*, which codes for linalool synthase, was highly expressed in ripening berries, whereas the gene for geraniol synthase peaked in expression in green berries and at the beginning of ripening [42]. With regard to the conversion of free terpenes to their bound forms, three monoterpenol *β*-D-glucosyltransferases—*Vvi*GT7, *Vvi*GT14 and *Vvi*GT15—were recently biochemically characterized [43, 44]. *Vvi*GT7 was demonstrated to mainly convert geranyl and neryl into their bound forms during grape ripening [43], whereas *Vvi*GT14 can glucosylate geraniol, *R, S*-citronellol, and nerol with similar efficiency, and *Vvi*GT15 prefers geraniol overnerol [44]. *Vvi*GT16, another uridine diphosphate glycosyltransferase (UGT), was also found to glucosylate monoterpenols and some short-chained and aromatic alcohols with low efficiency [44]. UGTs are responsible for the production of glycosyl-conjugated terpenes in grape berries. Although some important genes of the terpene biosynthetic pathway have been functionally identified and their expression patterns studied during grape berry development, it has not been entirely clear which genes play dominant roles in the accumulation of free and glycosidically bound terpenes in grape berries or which genes are easily affected at the transcriptional or translational level by climate factors. Answers to these questions will help to interpret the differences in terpene profiles in grape berries between regions and lay a basis for understanding the regulation of terpene biosynthesis.

Most wine-producing regions in China feature a continental monsoon climate with hot-wet summers and dry-cold winters. However, in northwest China, summer remains dry, with an annual rainfall of only 80–150 mm that is accompanied by strong sunshine and a large temperature difference between day and night. Relatively, east China has an annual rainfall of approximately 700 mm, concentrated in the summer-autumn seasons. These markedly different growing environments between the western and eastern regions of China cause differences in the qualities of mature grape berries and the flavors and sensory profiles of wines [19, 20, 45]. More recently, an investigation of the volatile profiles of Cabernet Sauvignon grapes grown in the northwest (Gaotai, Gansu province) and east (Changli, Hebei province) revealed that the variability of concentrations of C6 volatile compounds, 2- methoxy-3-isobutylpyrazine and damascenone strongly depended upon weather conditions during berry development [19]. Transcriptome comparisons of this variety in the two regions have also been extensively conducted [46]. Although the regional differences in flavor profiles of grapes and wines has always attracted Chinese researchers’ interest, terpene compounds receive insufficient attention, possibly because previous studies used non-aromatic varieties, such as Cabernet Sauvignon and Merlot, in which terpenes have fewer types and lower concentration.

The present study focused on Muscat blanc à Petit grains (*Vitis vinifera* L.) berries, a Muscat-type grape variety that is grown in two regions with distinct climates: Gaotai (GT) in Gansu Province in northwestern China and Changli (CL) in Hebei Province in eastern China. Winemakers originally noticed that this varietal wine made in the two regions presented somewhat different aroma performances. However, the terpene profiles and the relevant biosynthetic metabolism in grape berries have not yet been extensively researched. In this work, the concentrations of terpene volatiles (in both their free and glycosidically bound forms) and whole transcript-gene expression profiling were measured to identify the genes and potential transcript factors (TFs) that dominate or regulate the accumulation of terpenes in grape berries, and further to interpretate the differential accumulation of terpene volatiles observed between regions. The results from this work will promote our understanding of the complicated but important biosynthesis and regulation of terpenes, and offer some suggestions for local vineyard practices aimed to improve grape aromatic qualities.

## Results and discussion

### Comparison of free and glycosidically bound terpenes in the grapes between two regions

**Fig. 1:**
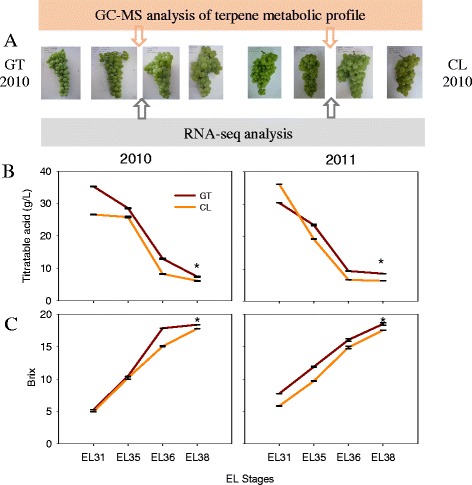
Sampling stages (**a**), titratable acidity (**b**), Brix (**c**), in the grapes at four developmental stages in two regions. Asterisk represents significant difference in Brix and titratable acid concentrations between CL and GT region at the EL38 stage (*p* < 0.05)

**Fig. 2:**
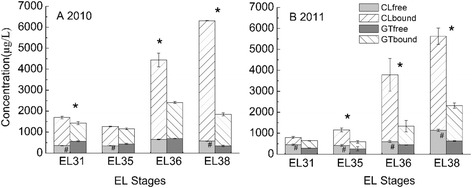
Change of total concentrations of free and glycosidically-bound volatiles. Columns indicate mean concentration (*n* = 3), and bars indicate standard error of the mean. Pound sign and asterisk represent significant difference of free and glycosidically-bound data between CL and GT region, respectively (*p* < 0.05). CL and GT is the abbreviation of Changli and Gaotai

**Fig. 3:**
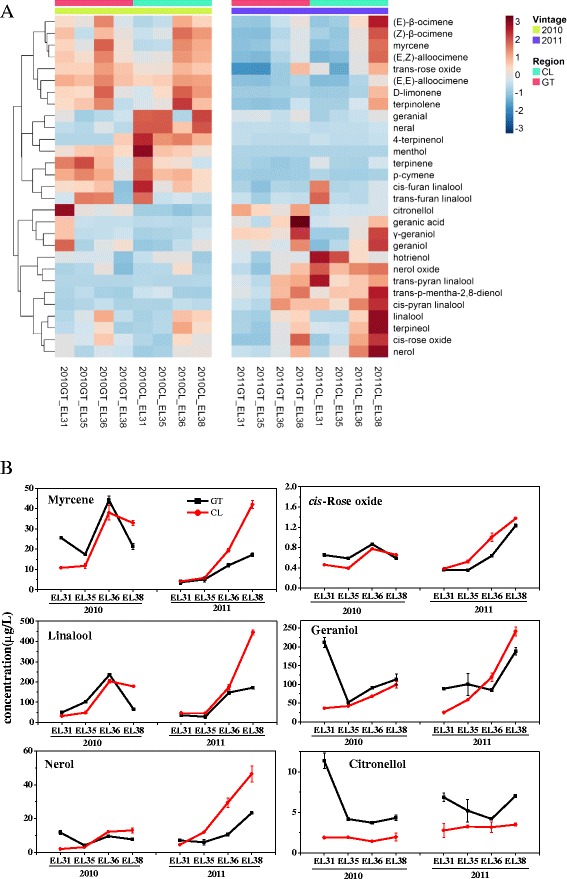
Profile of free volatiles in the grape berries in GT and CL regions. **a** A heatmap for the variation of free volatiles in the berries of two regions in 2010 and 2011. Each row represents an individual compound and each column represents an individual sample. The data was the mean of six values from each sample point. The data was normalized by rows used function “scale”. The topographycal colors are installed in deep red and deep blue, which depict relative concentration of terpenes from high to low. The color scale bar is shown at the right of the heat map. Dendrograms indicate the correlation between groups of terpenes; **b** Change in the concentration of main compounds in two regions in 2010 and 2011

Total soluble solid (°Brix) and titratable acid presented similar change patterns in developing grape berries between the two regions across two consecutive years. Nevertheless, the berries close to harvest (E-L 38) from GT contained significantly higher total soluble solid content and titratable acid compared with those from the CL region (Fig. 1). The total terpene concentration increased approximately 3-fold (CL) and 1.5 ~ 2-fold (GT), separately, along with ripening (Fig. 2). Statistically significant differences in the total concentrations of free and glycosidically bound terpenes were observed between CL and GT grapes, except for E-L 35 and E-L 36 in 2010. In particular, the difference in the concentration of the glycosidically bound form was much greater than the free form. Three evolutionary trends in the two-year time-course series could be clearly observed for free volatiles from the hierarchical heatmap clustering (Fig. 3a). In the first trend, volatiles such as geraniol, nerol, linalool, myrcene, *cis*-rose oxide generally presented an increase in their concentrations along with berry ripening (Additional file 1: Table S1A). Moreover, most compounds with the first evolutionary trend in mature grape berries had higher concentrations in the grapes grown in the CL region compared with the GT region. The compounds with the second evolutionary trend, such as terpinenols and *cis*/*trans*-furan linalool oxides, reached their highest levels at the pea-size period (E-L 31) or veraison (E-L 35) stage and subsequently reduced their levels in post-veraison grapes. At harvest, this group of volatile compounds did not display significant differences between the grapes from the CL and GT regions. The remaining compounds were grouped into the third evolutionary trend, including hotrienol, citronella and pyran linalool oxide. Their accumulation trends varied between regions and years. In the third group, hotrienol, a dehydrogenated form of linalool, displayed a downward trend as berry ripening processed, which was the opposite of the developmental accumulation of linalool. Among the detected free-form terpenes, linalool and geraniol had the highest concentrations, followed by nerol, mycene, citronellol and *cis*-rose oxide. Apart from citonellol, the other five terpenes presented higher concentration in mature grapes from the CL region than from the GT region (Fig. 3b). We must note that even in the same region, there was a great difference in the compound evolutionary trend between the two vintages. Because of this difference, we analyzed annual data instead of the mean of the two-year data. The findings indicate that the accumulation of free-from volatiles is easily altered by vintage.

**Fig. 4:**
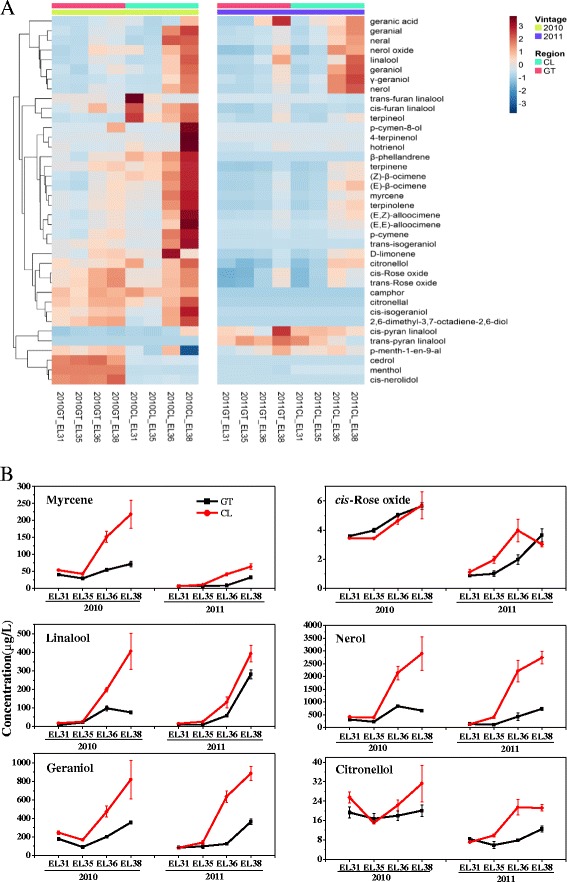
Profile of glycosidically-bound volatiles in the grape berries in GT and CL regions. **a** a heatmap of free volatiles in the berries of two regions in 2010 and 2011. Each row represents an individual compound and each column represents an individual sample. The data was the mean of six values from each sample point. The data was normalized by rows used function “scale”. The topographycal colors are installed in deep red and deep blue, which depict relative concentration of terpenes from high to low. The color scale bar is shown at the right of the heat map. Dendrograms indicate the correlation between groups of terpenes; **b** the concentration of main free-form compounds in the two regions in 2010 and 2011

Because most compounds accumulated from the veraison stage till ripe/harvest stage, glycosidically bound terpenes had high concentrations in mature berries (Fig. 4a). This developmental pattern was the same as those reported previously [4, 47–49]. Compared with the GT region, the concentrations of most bound volatiles were dramatically higher in the grapes from CL in both years. For example, glycosidically bound geraniol and nerol in the CL-produced grapes were 2 ~ 3-fold higher than in the GT-produced grapes (Fig. 4b). The glycosidically bound geraniol, nerol and linalool represent the three most abundant terpenes in Muscat Blanc à Petits Grains berries. In the present study, the differential accumulation of the three compounds between regions resulted in a large difference in the total concentration of terpenes, as shown in Fig. 2. Some other compounds, such as glycosidically bound forms of pyran linalool oxide (*cis*/*trans*), menthol and nerolidol, exhibited variable trends during berry development. However, these compounds all presented at low levels in grape berries. The proportion of free-form to glycosidically bound forms varied remarkably depending on the compounds themselves (Additional file 1: Tables S1A and B). We noticed that the linalool concentration was higher than the geraniol or nerol concentration in free-form terpenes, by contrast, the level of linaloyl glycoside was lower than geranyl and neryl glycoside, indicating that free-form linalool is less converted into the bound form. Neryl glycosides were the most abundant glycosidically bound monoterpene in Muscat Blanc à Petits Grains berries. The concentration of free-form citronellol was higher in the grapes from the GT region compared with the CL region, whereas citronellyl glycoside exhibited the opposite trend. Notably, some glycosidically bound terpenes presented significant differences in their concentrations between 2010 and 2011. For example, rose oxide (*cis*/*trans*), furan linalool oxide (*cis*/*trans*), citronellol, citronellal and hotrienol can be easily modified by oxidation or dehydrogenation, and ocimene, myrcene, terpinolene and limonene are produced by TPS-b subfamily enzymes. Hence, the difference in the aroma odor of vintage wines may be related to the production of these volatile compounds.

The concentrations of several aroma-related volatiles exceeded the sensorial threshold values in mature grapes, such as linalool, geraniol, myrcene and *cis*-rose oxide. This result indicates that these volatiles greatly contribute to the aromatic attributes of grape berries (Additional file 1: Table S1C). In addition, some glycosides, such as nerol, linalool and geraniol, also reached their respective thresholds, potentially contributing to the aromatic profile of wine (Additional file 1: Table S1C). The compounds that could have aroma contribution displayed different levels in the grapes from the CL and GT regions at the commercial mature stage (E-L38), thus causing distinctive aromatic senses.

### Expression profiles of terpene synthesis-related genes in the grapes

**Fig. 5:**
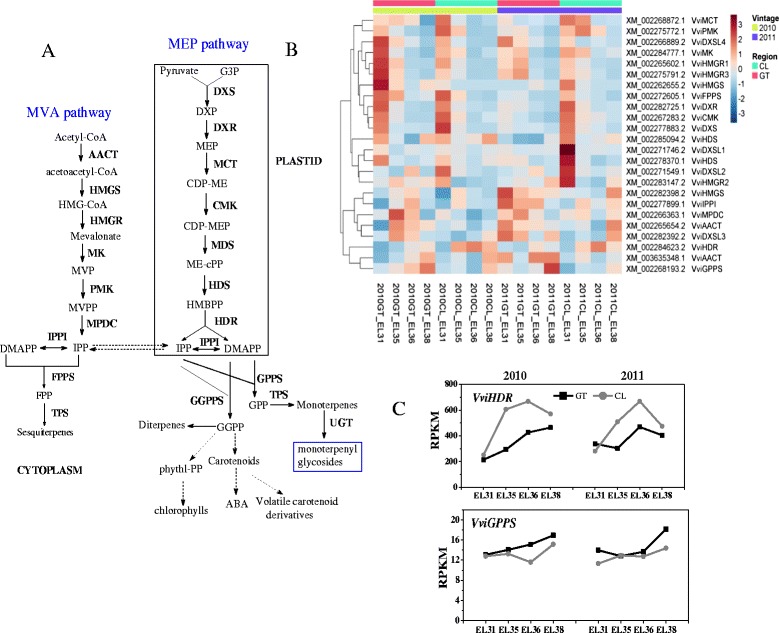
Expression profile of the genes in terpenoid backbone pathway in the grape berries. **a** Pathway of terpene biosynthesis in grape berries; The MEP pathway is localized in plastids, while the MVA pathway occurs in the cytosol. The following enzymes and metabolites are shown: G3P glyceraldehyde 3-phosphate, DXS 1-deoxy-D-xylulose-5-phosphate synthase, DXR 1-deoxy-D-xylulose 5-phosphate reductoisomerase, MEP 2-C-methyl-D-erythritol 4-phosphate, MCT 2-C-methyl-D-erythritol 4-phosphate cytidylyltransferase, CDP-ME 4-(Cytidine 5'-diphospho)-2-C-methyl-D-erythritol, CMK 4-(cytidine 5’-diphospho)-2-C-methyl-D-erythritol kinase, CDP-MEP 2-Phospho-4-(cytidine 5'-diphospho)-2-C-methyl-D-erythritol, MDS 2-C-methyl-D-erythritol 2,4-cyclodiphosphate synthase, ME-Cpp 2-C-Methyl-D-erythritol 2,4-cyclodiphosphate, HDS 4-hydroxy-3-methylbut-2-enyldiphosphate (HMBPP) synthase, HMB-PP (E)-4-hydroxy-3-methyl-but-2-enyl pyrophosphate, HDR 1-hydroxy-2-methyl- 2-(E)-butenyl-4-diphosphate reductase, IPP isopentenyl pyrophosphate, DMAPP dimethylallyl pyrophosphate, IPPI IPP-isomerase, GPPS geranyl pyrophosphate synthase, GPP geranylpyrophosphate; AACT acetoacetyl-CoA thiolase, HMGS 3-hydroxy-3-methylglutaryl synthase, HMG-CoA 3-hydroxy-3-methylglutaryl-CoA, HMGR 3-hydroxy-3-methylglutaryl-CoA reductase, MVA Mevalonate, MK MVA kinase, MVP Mevalonate-5-phosphate, PMK phospho-MVA kinase, MVPP Mevalonate-5-diphosphate, MPDC diphospho-MVA decarboxylase, MVPP mevalonate-5-pyrophosphate, FPPS farnesyl pyrophosphate synthase. **b** Transcription profile of the genes in the MEP and MVA pathway. Each row represents an individual gene and each column represents an individual sample. The data was normalized by rows used function “scale”. The topographycal colors are installed in deep red and deep blue, which depict relative expression abundances of genes from high to low. The color scale bar is shown at the right of the heat map. Dendrograms indicate the correlation between groups of genes. **c** Expression of two main genes in the MEP pathway

We first investigated the biosynthetic pathways of terpene precursors. Based on RNA-seq data, we quantified the transcript abundances of the genes required for the MVA and MEP pathways and the genes encoding isoprenyl diphosphate synthases, geranyl diphosphatesynthase (GPPS), farnesyl diphosphate synthase (FPPS) and geranylgeranyl diphosphate synthase (GGPPS). As shown in Fig. 5, the developmental expression patterns of these genes in the grapes were similar between 2010 and 2011.

The MEP pathway provides the precursors (IPP and DMAPP) for the synthesis of both monoterpenes and downstream carotenoids. The MEP pathway consists of seven chloroplast-localized enzymes [26, 50], of which six transcripts were expressed at four developmental stages in our experiment. Most of the genes were highly expressed at the early developmental stage (E-L31) and maintained a certain expression levels in the following process (Fig. 5b). Both *VviDXS* and *VviDXR* presented downward trends during grape maturation. DXSs are one of the main regulators of monoterpene biosynthesis in grapevine [35], of which *Vvi*DXS (XM_002277883.2) is the most important isoenzyme in grapes. In this study, *VviDXS* did not exhibit a statistically significant difference in transcript accumulation between the CL and GT-produced grapes. Additionally, the expression of *VviDXSL4* (XM_002266889.2) was significantly up-regulated in the grapes from the GT region compared with CL region at E-L35 stage, which was not in parallel with the production of monoterpenes. Therefore, *VviDXS* should not be a key gene responsible for the differential production of monoterpenes between the CL and GT regions. By contrast, *VviHDR* (XM_002284623.2, the final enzyme of the MEP pathway) could be a predominantly involved gene. As shown in Fig. 5c, the expression of *VviHDR* increased as grape development proceeded, and the increment in the CL-produced grapes was much greater than that in the GT-produced grapes, which highly paralleled with the accumulation of monoterpenes observed in the two regions and two vintages. The expression of *VviGPPS* (XM_002268193.2) increased slightly as berry matured, but didn’t show statistical significance in the abundance between the two regions.

IPP and DMAPP are also produced through the cytoplasmic MVA pathway. This pathway consists of six enzymes, for which all transcripts were observed in each of the four developmental stages. Except for the two transcripts encoding acetyl-CoA acetyltransferases (AACT, XM_002265654.2 and XM_003635348.1), the other four exhibited downward trends with berry maturation. For example, two of the three transcripts encoding isoforms of 3-hydroxy-3-methylglutaryl-coenzyme A reductase (HMGR) and the transcript encoding FPP synthase generally decreased during berry development. HMGR is a rate-limiting enzyme in the MVA pathway [51, 52]. However, in this study, the three *VviHMGRs* in the berries of the GT region were expressed higher than those from the CL region at E-L35 (Additional file 1: Table S2), whereas only a few sesquiterpenes compounds were identified in the berries at that stage, suggesting that the expression of *VviHMGRs* did not entirely correlate with the production of sesquiterpenes in cytoplasm.

**Fig. 6:**
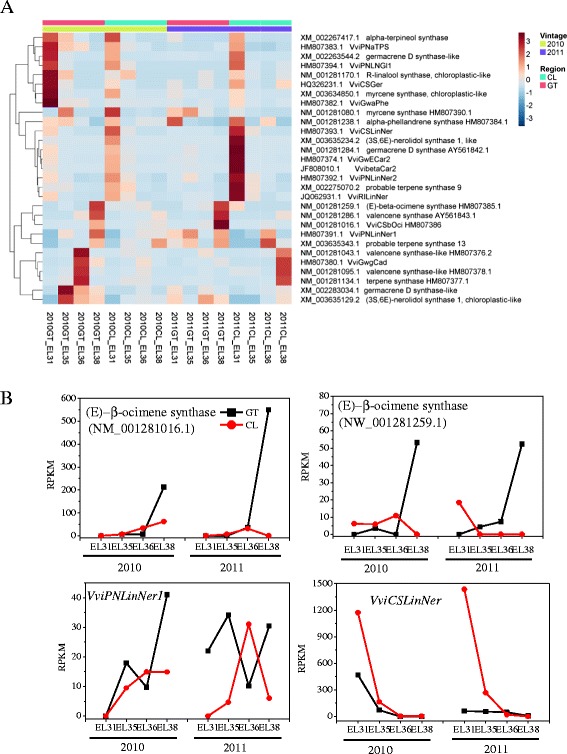
Expression profile of the genes coding for terpene synthases (*Vvi*TPSs) detected in this study; **a** transcription expression profile of terpene synthases detected in this study. Each row represents an individual gene and each column represents an individual sample. The data was normalized by rows used function “scale”. The topographycal colors are installed in deep red and deep blue, which depict relative expression abundances of genes from high to low. The color scale bar is shown at the right of the heat map. Dendrograms indicate the correlation between groups of genes. **b** Expression of the four terpene synthases in the two regions in 2010 and 2011

*VviTPSs* are a large gene family responsible for the convertion of GPPS into a variety of terpenes. At present, sixty-seven *VviTPS* isogenes were identified from our RNA-seq data. Based on the sequence homology to the functionally characterized *TPSs* in the NCBI nr database, these genes were grouped into the TPS-a, TPS-b and TPS-g subfamilies. Cluster analysis was applied to identify genes with similar expression patterns. Sesquiterpenes are produced through the members of the TPS-a subfamily from farnesyl pyrophosphate (FPP) that is formed via the MVA pathway in the cytoplasm. We identified 20 transcripts encoding putative TPS-a enzymes, some of which were annotated by NCBI as valencene synthases-like, germacrene synthases-like or (*E*)-beta-caryophyllene synthases. In our analysis, however, ten of the 20 TPS-a transcripts were detectable only at one or two developmental stages of grapes, so were not assigned to the heatmap cluster. The other 10 transcripts exhibited detectable levels across all four developmental stages (Table 1). Of these 10 transcripts, four were expressed primarily in young berries (HM807374.1 (NM_001281275.1), XM_002263544.2, NM_001281284.1 and JF808010.1), whereas the other six genes were expressed specifically in mature berries (XM_002283034.1, HM807380.1, NM_001281095.1, NM_001281043.1, NM_001281134.1, and NM_001281286.1) (Fig. 6a). Moreover, the expression of the gene (NM_001281134.1/ HM807377.1) coding for germacrene D synthase presented an upward trend in the mature process of grapes. (+)-Valencene synthase (NM_001281286.1, AY561843.1/FJ696653.1, *Vvi*ValCS) is a key enzyme of sesquiterpene biosynthesis and contributes greatly to the production of aromatic volatiles in both aromatic white and non-aromatic grapevine cultivars [40, 53]. Although *VviValCS* had a high expression level in mature berries in this study, no detectable sesquiterpenes were present in the corresponding berries. In contrast, only a few sesquiterpenes, such as α-muurolene, α-calacorene and cedrol, were qualitatively identified in green berries (they could not be quantified, data not shown). According to the inconsistence between transcript abundance and metabolite concentration, it is inferred that *Vvi*ValCS was not associated with the production of sesquiterpenes in this grape variety. The biochemical significance of high *VviValCS* transcript level in mature berries will also be an issue of ongoing investigation in our future research.

**Table 1:** Terpenoid pathway transcripts

Encoded protein description	Cluster	RefSeq accession(s)
3-hydroxy-3-methylglutaryl-coenzyme A reductase(HMGR)	Decreased	XM_002265602.1,XM_002275791.2
	Stable expression	XM_002283147.2
Farnesyl pyrophosphate synthase(FPPS)	Decreased	XM_002272605.1
TPS-a (sesquiterpene synthase, 20)	NC	XM_002275022.1,XM_002275315.1,XM_002282452.1,XM_002283308.1,HM807375.1,XM_003635502.1,NM_001281075.1,NM_001281086.1,NM_001281099.1,NM_001281272.1
	Decreased(young berry)	HM807374.1(NM_001281275.1),XM_002263544.2,NM_001281284.1,JF808010.1
	Increased(ripe berry)	XM_002283034.1, HM807380.1, NM_001281095.1,NM_001281134.1,NM_001281043.1, NM_001281286.1
1-deoxy-D-xylulose-5-phosphate synthase(DXS)	Stable expression	XM_002277883.2
1-deoxy-D-xylulose-5-phosphate synthase, chloroplastic-like		XM_002271746.2,XM_002271549.1,XM_002282392.2,XM_002266889.2
1-deoxy-D-xylulose 5-phosphate reductoisomerase (DXR)	Stable expression	XM_002282725.1
4-hydroxy-3-methylbut-2-enyldiphosphate reductase (HDR)	Increased	XM_002284623.2
geranyl diphosphate synthase (GPPS)	Stable expression	XM_002268193.2
TPS-b (monoterpene synthase, 25)	NC	XM_002266772.1,XM_002267425.2,XM_003634831.1,HM807387.1,HM807388.1,AY572986.1,AY572987.1,NM_001281254.1
	Decreased(young berry)	XM_002275070.2,XM_002267417.1,XM_003634850.1,HM807382.1,HM807383.1,NM_001281170.1,NM_001281238.1,NM_001281080.1
	Increased(ripe berry)	NM_001281016.1(HM807386.1)
	ND	XM_002267123.1,XM_003634833.1,XM_003634834.1,XM_003634835.1,XM_003634837.1,XM_002266983.2,XM_003634854.1
	Different expression	NM_001281259.1(HM807385)
TPS-g (monoterpene synthase, 21)	Decreased(young berry)	HQ326231.1,HM807392.1,HM807393.1,HM807394.1,XM_003635234.2
	NC	HM807395.1,HM807396.1,HM807397.1,HM807398.1,HM807399.1,XM_003635120.2, XM_003635365.2
	Increased(ripe berry)	HM807391.1,XM_003635129.2,XM_003635343.1
	ND	XM_003633271.1,XM_003635121.1,XM_003635122.1,XM_002270071.2,XM_003635233.1,XM_003635244.1

*NC* expressed at a certain stage but not clustered in heatmap, *ND*, not included in the current NCBI RefSeq mRNA database

Monoterpenes are produced by the members of the TPS-b and TPS-g subfamily (Table 1). Of the 25 putative TPS-b genes (Table 1), seven genes were absent in the current NCBI RefSeq mRNA database (updated: 2014-12-10) and excluded in the following analyses. Of the remaining 18 genes, eight were detected at only one or two stages in this investigation, whereas the other 10 exhibited detectable expression levels throughout grape development (Table 1). Eight of these 10 transcripts exhibited a downward trend during grape development (XM_002275070.2, XM_002267417.1, XM_003634850.1, HM807382.1, HM807383.1, NM_001281170.1, NM_001281238.1 and NM_001281080.1), and one transcript encoding (*E*)-beta-ocimene synthase (NM_001281016.1 in NCBI/ HM807386 in Martin et al., [39]) was expressed mostly in mature grapes. This gene expression was up-regulated in the berries of the GT region compared with the CL region at E-L 38 stage (Fig. 6b), which was not according with the accumulation of ocimenes. The present result was also consistent with another report [42]. Accordingly, the expression of this transcript for (*E*)-beta-ocimene synthase (NM_001281016.1) likely affects the production of ocimenes in the two investigated regions to a large extent. Another transcript encoding (*E*)-beta-ocimene synthase (NM_001281259.1 in NCBI, HM807385 in Martin et al. [39]) displayed different expression patterns in the grapes from the two regions in the two vintages. In detail, this gene expression in the GT grapes presented an upward trend in both of vintages. With regard to the CL grapes, its expression tended to rise from E-L 31 to E-L 36, and afterwards dropped at E-L 38 in the 2010 vintage, but the transcript was detected only at E-L 31 of the 2011 vintage (Fig. 6b). So we infered that the expression of this gene was not closely associated with the production of ocimene in mature berries. Based on the developmental expression pattern, two α-terpineol synthases, VviTer1 (AY572986.1) and VviTer2 (AY572987.1), were also considered not to be responsible for monoterpene accumulation in these Muscat Blanc a Petits Grains grapes because they displayed low expression levels that were only detected at a few stages. Conversely, the two transcripts annotated as alpha-terpineol synthase (XM_002267417.2) and myrcene synthases (XM_003634850.1) exhibited high abundances (XM_002267417.2 with RPKM > 900; XM_003634850.1 with RPKM > 5800). Accordingly we deduced that these two myrcene synthases were involved in the high accumulation of monoterpenes in this grape variety.

Twenty-one transcripts were grouped into the TPS-g subfamily (Table 1). Among them, six had been removed from the current RefSeq mRNA database (2014-12-10 updated). The TPSs of this subfamily exclusively produce acyclic terpene alcohols. 10 *TPS-g* genes had been biochemically characterized by Martin et al. [42]. Of these functionally known *TPS-g* genes, five genes (HQ326231.1, HM807392.1, HM807393.1, HM807394.1 and XM_003635234.2) presented downward trends in the transcript production as berry ripening progressed (Fig. 6a), which was inconsistent with the accumulation of free monoterpene alcohols in this variety. This result also verified the previous finding that the expression of most *TPSs* did not entirely correlate with the production of terpene volatiles in grape berries [54, 55]. There may be regulation at the translational level, such as protein amount, enzyme activity or post-translational modifications. Notably, among the seven genes that have been demonstrated to be responsible for linalool synthesis *in vitro* [39], only *VviPNLinNer1*(HM807391.1) expression presented an upward trend with berry development (Fig. 6b), which paralleled with the accumulation of linalool (Fig. 4b). In Moscato Bianco grapes (a Muscat variety), *VviPNLinNer1* also displayed a similar developmental expression pattern [42]. The expression trend of *VviPNLinNer1* was quite different in 2011 GT-produced berries. With regard to the comparison between two regions, the expression of *VviPNLinNer1* at the E-L38 stage was up-regulated about 2.5-fold in the GT grapes in comparison to the CL grapes (Additional file 1: Table S2), whereas the concentration of linalool in matue grapes of GT was significantly lower (Fig. 4b). Evidently the differential accumulation of linalool between the grapes of both regions did not simply depend on the expression of this gene alone. *VviCSLinNer* (HM807393.1) was highly expressed at the E-L31 stage and rapidly declined at subsequent stages (Fig. 6b). The transcript abundance of this gene in the CL grapes was nearly 4-fold higher than that in the GT grapes at E-L 31 stage (Additional file 1: Table S2) when the CL grapes had higher concentration of bound linalool (Additional file 1: Table S1B). This implies that the expression of *VviCSLinNer* is likely region-dependent. Zhu et al. also observed that *VviCSLinNer* was highly expressed in the early developmental stages of Gewurztraminer grapes [56]. By contrast, Martin et al. observed that *VviCSLinNer* had an expression peak at veraison in Gewurztraminer grapes [11]. In our study, Three genes encoding for geraniol synthase: *VviCSGer* (HQ326231.1), *VviGwGer* (HM807398.1), and *VviPNGer* (HM807399.1) were also uniquely expressed at the green stage (E-L31 and E-L35), indicating that the expression of these genes is developmentally specific.

In addition, five genes that are currently annotated by NCBI as nerolidol synthases (XM_003635120.1, XM_003635129.1, XM_003635234.1, XM_003635365.1, and XM_003635343.1), two transcripts (XM_003635129.1 and XM_003635343.1) presented increasing expression levels along with the development of the grape berry, with one (XM_003635129.1) expressed higher in the berries of the GT region than of the CL region. Another transcript (XM_003635234.1) had higher levels in the berries of the CL region compared with the GT region, suggesting that the accumulation of nerolidol in both regions should be dependent on the expression of this gene expression to a large degree.

### Genes corresponding to monoterpenol glucosyltransferases

**Fig. 7:**
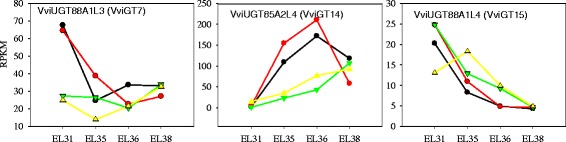
Expression profile of three genes corresponding to monoterpenol glucosyltransferases

Monoterpenol *β*-D-glucosyltransferases (GTs) are responsible for the conversion of free terpenes into their glycosidically bound form. For wine grapes, this enzyme is particularly important because free-form monoterpenes in grapes can be easily sent out to the atmosphere once they are produced, and the level of glycosidically bound monoterpenes, a storage form of volatiles in grapes, actually reflects the potential aromatic quality of grapes and wines. GTs are a large gene family that has not yet been clearly understood. Recently, monoterpenol *β*-D-glucosyltransferases (GTs) have been isolated from different grape varieties and biochemically characterized; they demonstrate high activity to geraniol, nerol and citronellol and contribute to the production of their glucosides during grape ripening [43, 44]. In this study, *VviUGT88A1L3* (*VviGT7* in Bönisch et al., [43]) showed similar expression trends in the two vintages with regard to the same region-produced grapes, so did *VviUGT85A2L4* (*VviGT14* in Bönisch et al., [44]) (Fig. 7). As for the grapes of CL region, *VviUGT88A1L3* (*VviGT7*, XM_002276510.2) was highly expressed at the pea-size stage (E-L31), much higher than that in GT grapes, and there was a sharp declining from E-L 31 to E-L 35 (in 2010 vintage) or E-L 36 stage (in 2011), followed by an increase at the E-L38 stage. This expression pattern was consistent with that observed in other Muscat grapes [43]. The cumulative expression of this gene was positively correlated with the concentrations of geranyl and neryl glucosides (Additional file 1: Table S3). Moreover, the expression of *VviUGT88A1L3* at the E-L 31 stage was highly up-regulated in the CL region relative to the GT region. *VviUGT88A1L3* expression should partially contribute to the accumulation of geranyl and neryl glucosides during grape ripening. *VviUGT85A2L4* (*VviGT14*, XM_002285734.2) expression in the berries of the CL region generally increased during E-L 31 to E-L 36 and decreased at the E-L 38 stage but increasingly increased in expression along with grape berry development in the GT region. This gene expression was significantly up-regulated in the CL-produced grapes relative to the GT-produced grapes. According to the data acquired in the grapes of two regions and two vintages, the expression of *VviUGT85A2L4* strongly positively correlated with the concentrations of geranyl, neryl and linayl glucosides in Muscat Blanc à Petits Grains berries (r = 0.93, 0.94, 0.86, respectively, *p* < 0.05; Additional file 1: Table S3). From the significant difference in *VviUGT85A2L4* transcript abundance between the berries of the two regions, it is inferred that *VviUGT85A2L4* could be environmentally induced, and differential accumulation of glycosidically bound geranyl and neryl between the regions should largely depend on the expression of this gene. The expression of *VviUGT88A1L4* (*VviGT15*, XM_002281477.2) gradually decreased in developing berries, apart from the higher expression in 2011-vintage GT grapes at the E-L 35 stage than at the E-L 31 stage. Moreover, this gene did not exhibit significant difference in the transcript abundance between the regions. Therefore it is thought that *VviUGT88A1L4* is not associated with the differential accumulation of glycosidically-bound terpenes across the two regions. As for *VviUGT85A2L5* (*VviGT16*, XM_002263122.1), its transcript was not detected in this study. Bönisch and his colleagues also found that *VviUGT85A2L5* has little involvement in the glycosylation of these compounds in *Vitis vinifera* grapes [44].

To identify additional candidate *Vvi*UGTs that act in the synthesis of glycosidically bound terpenes in grape berries, we adopted K means clustering analysis to cluster the expression patterns of 147 *VviUGTs* corresponding to UDP-glycosyltransferases (UGTs) in our RNA-seq data (Additional file 2: Figure S1). A total of 32 *VviUGTs* in clusters 1, 2 and 3 exhibited upward trends in expression parallel with the production of glycosidically bound terpenes (Additional file 2: Figure S1A, detailed information of the selected genes is provided in Additional file 1: Table S4A). A phylogenetic tree was conducted based on the amino acid sequences of the 147 *Vvi*UGTs. These genes were divided into several groups (Additional file 2: Figure S1B, detailed information of the selected genes is provided in Additional file 1: Table S4B). Twenty-four sequences displayed high similarity with known terpene GTs (VviGT7/ VviGT14/ VviGT15/ VviGT16). Combining the results of the K means analysis with the sequence similarity analysis; we speculated that these four transcripts should be putative monoterpenol glucosyltransferases. According to the grapevine gene naming system recommended by Grimplet et al. [57], they were named as *VviUGT88A1L1* (XM_002276679.2), *VviUGT*86A1L (XM_002276822.1), *VviUGT85A1L1* (XM_002285742.2) and *VviUGT85A1L3* (XM_002268601.2). The four genes were all increasingly expressed as grapes ripen. The transcript accumulation of *VviUGT85A1L1* and *VviUGT88A1L1* was positively correlated with the production of geranyl, neryl and linaloyl glucosides in Muscat Blanc à Petits Grains berries (Additional file 1: Table S3). Furthermore, *VviUGT85A1L1* was up-regulated at the E-L36 stage in the CL region relative to the GT region, which was consistent with the accumulation of geranyl, neryl and linayl glucosides in berries. As a result, the expression of *VviUGT85A1L1* was probably related to differential accumulation of these bound compounds across the two regions. Further biochemical characterization is necessary to better understand the mechanisms of these putative glucosyltransferases.

In summary, based on the associations between the transcript accumulations and the production of final metabolites, we identified some genes that possibly dominate the differential accumulation of free-form and/or glucosidically bound monoterpenes in the CL and GT regions, such as *VviHDR* (XM_002284623.2)*, VviCSLinNer* (HM807393.1), a nerolidol synthase gene (XM_003635234.1), *VviGT14* (XM_002285734.2) and *VviUGT85A1L1* (XM_002285742.2)*.* Regardless of the effect of vintage, these genes were all significantly differentially expressed between the regions. In addition, other regionally differentially expressed genes (DEGs) were also identified, including *VviDXS5* (XM_002266889.2), three *VviHMGR* genes (XM_002265602.1, XM_002283147.2 and XM_002275791.2) and 8 *VviTPSs*. However, the accumulation of their transcripts was not strongly positive correlated with the production of final terpene metabolites.

### Co-expression network analysis of transcription factors (*TFs*) and differentially expressed genes (DEGs)

**Fig. 8:**
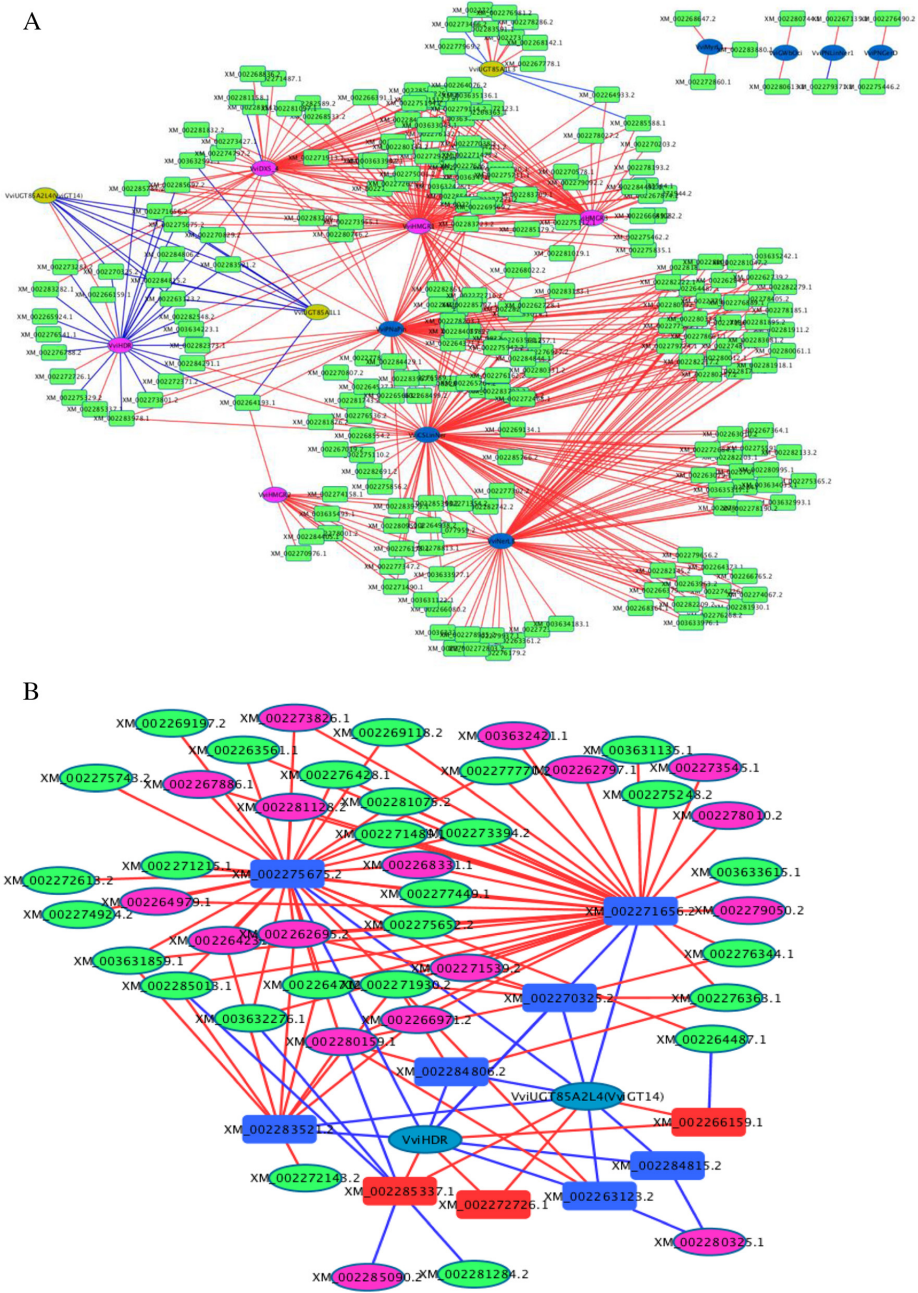
**a** Co-expression network analysis for the differentially-expressed structural genes and candidate transcription factor (TF) genes. The TFs listed in the plot have a high correlation coefficient (≥|0.8|) with structural genes in terms of transcript accumulation. Structure genes are represented as circle nodes. Different colors are used for the various gene categories: pink for genes in terpene precursory pathway, blue for terpene synthase genes, yellow for glucotransferase genes. TFs are represented as rectangle nodes, and TF gene ID is shown in the tectangle. The annotation of all genes and TFs in this network is listed in Additional file 1: Table S6. **b** Co-expression network analysis for structural genes, candidate TF genes and ripening-associated genes. In this network, structural genes were *VviHDR* and *VviUGT85A2L4* (*VviGT14*) that potentially dominate differential accumulation of terpenes in the grapes between the GT and CL regions; TFs in plot B are those that positively (in red rectangle) and negatively (in blue rectangle) co-expressed with both *VviHDR* and *VviUGT85A2L4* (*VviGT14*); the ripening-associated genes listed in plot B have over 0.8 of the correlation coefficient absolute value with TF genes in terms of transcript accumulation. Pink oval indicates the genes related to ABA biosynthesis and signal transduction, and green oval represents the genes related to ethylene biosynthesis and signal transduction. In plots A and B, lines connecting two nodes represent significant correlation: red means a positive correlation and blue means a negative correction

To identify potential transcription factors (TFs) that regulate these DEGs, we performed network analysis of the correlations between the expression levels of various *TFs* and the DEGs. Based on the annotated grape genome, we first selected 725 transcription factors (TFs) of different classes in the present database. Pearson correlation coefficients were calculated with respect to each pair of variables (structural genes vs. *TFs*) across the profiles at various developmental stages. DEGs and *TFs* with high correlation coefficients (absolute value > 0.8) were connected by a line to construct a correlation network module. Co-expression between DEGs and *TFs* was additionally visualized in Fig. 8a.

In recent years, some TFs of the MYC, WRKY, AP2, AP2/ERF and MYB families have been reported to be involved in the transcriptional regulation of terpene synthesis genes in other plants, such as *Catharanthus roseus*, *Arabidopsis* and *Solanum lycopersicum* trichomes [58–62]. Most of these identified TFs control the promoters of sesquiterpene synthase genes. In this study, some members of these TF families were also positively or negatively co-expressed with DEGs, including genes not only involved in the MEP and MVA pathways but also in the synthesis of free and glucosidically bound monoterpenes. For example, AP2/ERF/B3 (XM_002276456.1) strongly positively correlated with *VviDXSL4* (XM_002266889.2), *VviHMGRs* (XM_002265602.1 and XM_002275791.2) and *VviPNaPin* (HM807384.1) transcript accumulation with coefficients of 0.84, 0.90, 0.80, 0.87, respectively (Additional file 1: Table S5); HMGR is an enzyme in the biosynthetic pathway of sesquiterpenes (Fig. 5). In *Artemisia annua*, two AP2/ERF family transcription factors (ERF1 and ERF2) up-regulated the expression of the gene encoding amorpha-4,11-diene synthase (a sesquiterpene synthase) [60]. Moreover, we observed that an ethylene-responsive TF (XM_002267364.1, *VviCRF4*), six AP2/ERFs, forty-five ERFs, four MYCs, twenty WRKYs and nine MYBs highly co-expressed with several *VviTPSs,* such as *VviCSLinNer* (HM807393.1) and nerolidol synthase-like gene (NM_001280966.1/HM807396.1) (Additional file 1: Table S5), suggesting that these TFs could potentially activate the promoters of the above structural genes.

The transcriptional regulation of monoterpenol glycosyltransferases (GTs) recently identified in grapes is not yet understood. This co-expression network analysis revealed that many TFs strongly negative correlated with transcript accumulation of *VviUGT85A2L4* (*VviGT14)* and the other two glucosyltransferase genes, *VviUGT85A1L1* (XM_002285742.2) and *VviUGT85A1L3* (XM_002268601.2) (Fig. 8a). These potential TFs included the members of the bHLH, HD-Zip, GATA, NF-YC, NF-YB families that respond to light [63, 64]. Notably, *Vvi*ERF3L (XM_002285337.1), *Vvi*GATA5L (XM_002272726.1) and *Vvi*GT-2 L (XM_002266159.1, a trihelix TF), positively co-expressed with *VviUGT85A2L4* (*VviGT14*). The trihelix TF (XM_002266159.1) transcript increasingly accumulated with grape ripening and responded to the production of glycosidically bound monoterpenes. In the work of Kaplan-Levy and his colleagues, the trihelix family TFs were found to respond to light, stress and development [65]. Based on our present finding, we suggest that the trihelix TF (XM_002266159.1, *Vvi*GT-2 L) could be involved in the regulation of glycosidically bound monoterpene biosynthesis. Additionally, one MYB TF (XM_002265012.1, *VviMYBA2*), two WERK TFs (XM_002277846.2 and XM_002284930.1) and two ERF TFs (XM_002285337.1 and XM_002263269.2) also positively co-expressed with *VviGT1,* with a correlation coefficient of approximately 0.7.

Based on this co-expression analysis, the functions of some TFs were predicted. For example, *VviCAMTA4L* (XM_002270829.2, a calmodulin-binding TF) had a strong positive correlation with *VviDXSL4* (XM_002266889.2), *VviHMGR1 (*XM_002265602.1), *VviHMGR2* (XM_002275791.2), and *VviPNaPin* (HM807384.1) in terms of transcript accumulation, but was highly negative correlated with *VviHDR* (Additional file 1: Table S5). CAMTAs (calmodulin binding transcription factors) link environmental cues with phytohormone-dependent growth responses. *Arabidopsis* CAMTAs are induced by both biotic and abiotic stresses and respond differentially and rapidly (within <15 min) to heat stress, cold stress, high salinity, drought, UV radiation, mechanical wounding, phytohormones (ethylene and ABA) and signal elicitors, such as methyl jasmonate (MJ) and salicylic acid (SA) [66, 67]. This study also revealed that *VviCAMTA4* could respond to distinctive climates of the CL and GT regions at the transcriptional level and regulate the expression of monoterpene synthesis-related genes. Additionally, heat shock transcription factors (Hsf) have been shown to participate in the regulation of heat responses in berries [68]. In this study, five members of the Hsf family also displayed high co-expression with *VviPNLinNer1* and *VviCSLinNer*, two nerolidol synthase-like genes (NM_001280966.1/HM807396.1; XM_003635234.1). Recently, PIF5, a basic helix-loop-helix (bHLH) transcription factor, was found to regulate the transcription of MEP pathway genes and function as an IPP-metabolism enhancer [69]. In the present prediction, both PIF3 (XM_002276162.2) and PIF1 (XM_002263361.2) exhibited strong co-expression with *VviCSLinNer* (HM807393.1), *VviNerL8* (XM_003635234.1) and *VviPNaPin* (HM807384.1). Therefore, PIFs (such as PIF3 and PIF1) are also probably involved in the regulation of terpene biosynthesis downstream pathway in grapes.

To further understand which TFs potentially contribute to regionally differential accumulation of terpenes, we identified the differentially-expressed *TFs* in grapes of the same developmental stage across two regions. The result showed that there were different candidate TFs at four developmental stages of grapes (Additional file 1: Table S6). At the E-L 31 and E-L 38 stages, except for the gene coding for a homeobox-leucine zipper protein HOX3-like (XM_002280613.2), the other candidate *TFs* all had significantly lower expression levels in the grapes of the GT region than in the CL grapes and most positively co-expressed with the DEGs (Additional file 1: Table S6). Conversely, at the E-L 34 and El-35 stages, most of the candidate *TFs* were transcriptionally up-regulated in the CL-produced grapes relative to the GT grapes. Notably, HD-zip (XM_002271656.2, a homeodomain associated leucine zipper protein) negatively correlated with both *VviHDR* and *VviUGT85A2L4* (*VviGT14*) levels with respect to transcript accumulation but was significantly up-regulated in the grapes of the GT region relative to the CL grapes at the E-L 35 stage. The HD-Zip proteins have been considered important candidates to activate developmental responses to altering environmental conditions [70, 71]. Therefore, it is possible that HD-zip (XM_002271656.2) controls the expression of *VviHDR* and *VviUGT85A2L4* (*VviGT14*) to profoundly affect differential production of terpenes in the GT and CL regions.

Our gene co-expression network analysis provides a possibility for the prediction of potential transcription factors. However, further experiments should be conducted to verify whether these putative TFs can activate the promoters of structural genes in the terpene biosynthetic pathway in grapes. From the well-studied cases of transcriptional regulation in other plants, such as *Catharanthus roseus* and *Arabidopsis*, it has been clearly illustrated that transcriptional regulation usually involves a network of TFs. The present network analysis gives us some research ideas on the regulation of terpene biosynthesis in grape berries.

### Ripening hormone-associated genes and their co-expression network

Both abscisic acid (ABA) and ethylene have been demonstrated to respond to grapevine growing environments and trigger grape berry ripening [72–74]. Chinese grape planters have noticed that grape berries generally have shorter duration at both the veraison and maturation stages in the GT region of western China compared with the grapes in the CL region of eastern China, as shown in Table 2. Herein, we were concerned about the genes involved in the biosynthesis and signaling response of ABA/ethylene. Based on the RNA-seq data in this study, we identified differentially expressed genes (DEGs) at a certain phenological phase corresponding to the GT and CL regions (Additional file 1: Table S7). Most of these genes were transcriptionally up-regulated at the E-L 31 and E-L 35 stages in the berries of the GT region relative to the CL region, indicating that grape ripening in the GT region starts earlier than in the CL region. For example, some genes associated with ABA biosynthesis/response were differentially expressed between the two regions. Phytoene synthase (XM_002271539.2, *VviPSY*) and capsanthin/capsorubin synthase (XM_002273826.1) are two key enzymes in ABA biosynthesis. The expression of these two genes and two ABA-response transcripts (XM_003631566.1, XM_002280159.1) was significantly up-regulated in the GT region at the beginning of veraison (E-L 35) (Additional file 1: Table S7). Similarly, many of the genes that are required for ethylene biosynthesis/signal response were also expressed significantly higher in the berries of the GT region compared to the CL region at the E-L 35 stage. A previous report demonstrated that ethylene largely produces before veraison in ‘Cabernet Sauvignon’ berries [74]. Another recent study also identified that ethylene is involved in triggering berry ripening, and an ethylene peak precedes the ABA peak in Muscat Hamburg berries [75]. In the GT region of western China, shorter veraison and ripening periods of grape berries (Table 2) can be interpreted by the difference in ABA- and ethylene-related transcriptome observed between the GT and CL regions. Additionally, ABA is also a stress-stimulated signal, and this hormone rapidly accumulates in the berries in response to water deficit and low temperature [18, 76]. Compared with the CL region, the GT region had less rainfall, stronger sunshine and larger day-night temperature differences (Table 2), which could promote the expression of ripening-related genes, such ABA and ethylene-associated genes, thereby accelerating the process of berry maturity.

**Table 2:** The meteorological index and grape development in CL and GT

	Days		RAD(kj/m2)		GDD		Sunshine duration (h)		Rainfall (mm)		Temperture difference between day and night(°C)	
2011	CL	GT	CL	GT	CL	GT	CL	GT	CL	GT	CL	GT
Flowering	13	7	31508	12253	127.70	99.90	132.00	64.10	3.50	0.00	11.39	16.10
Berry development	48	53	110124	81898	575.30	739.00	250.70	525.30	181.10	25.70	6.42	14.60
veraison	24	17	46340	28011	353.40	180.70	101.60	126.90	342.70	48.80	5.60	12.70
Ripening	36	25	69717	38491	394.00	191.00	290.70	221.70	28.00	6.40	9.73	13.30
Total	121	102	257689	160653	1450.40	1210.60	775.00	938.00	555.30	80.90	8.29	14.18
2010	CL	GT	CL	GT	CL	GT	CL	GT	CL	GT	CL	GT
Flowering	5	6	11537	17608	47.40	87.10	55.80	79.30	0.00	0.00	10.40	17.60
Berry development	51	62	87562	138144	671.80	884.10	320.70	612.60	162.90	32.60	6.60	14.30
Veraison	20	16	30430	34442	232.10	178.80	106.90	168.20	219.50	9.40	6.70	15.82
Ripening	34	25	43105	34589	363.80	142.10	182.40	161.20	159.70	65.70	7.90	11.50
Total	110	109	172634	224783	1367.00	1292.10	665.80	1021.30	542.10	107.70	7.20	14.81

To explore the effect of grape maturation rate on the accumulation of terpenes, we constructed a co-expression network to visualize the correlations among the genes of three categories. The first category included *Vvi*HDR and *VviUGT85A2L4* (*VviGT14*). Base on a highly positive correlation between gene transcript abundance and terpene concentration, it is proposed that *Vvi*HDR and *VviUGT85A2L4* (*VviGT14*) potentially dominate the regionally differential accumulation of terpenes in the grapes. The second one consisted of TF genes that have a high correlation coefficient (≥|0.8|) with both *Vvi*HDR and *VviUGT85A2L4* (*VviGT14*). And the third one was composed of ABA/ethylene-related genes (Fig. 8b). Seven TFs had a strongly negative correlation with both *VviHDR* and *VviUGT85A2L4* (*VviGT14*). These TFs coded for XM_002275675.2 (ICE1-like TF), XM_002263123.2 (TF HBP-1b(c1)), XM_002270325.2 (GATA TF), XM_002271656.2 (Zip family TF), XM_002283521.2 (IIE subunit 2), XM_002284806.2 (NF-YB8 TF) and XM_002284815.2 (NF-YC9 TF). The genes for XM_002275675.2 (ICE1-like TF) and XM_00227165.2 (Zip family TF) positively correlated with many ABA/ethylene-related genes in terms of transcript accumulation. Therefore, grape ripening acceleration probably causes the down-regulation of critical genes in the terpene biosynthetic pathway, ultimately resulting in decreased metabolite production. This suggestion was also supported by the following correlation. Three TFs coding for XM_002285337.1 (ERF003), XM_002266159.1 (trihelix transcription factor GT-2) and XM_002272726.1 (GATA transcription factor 5) positively co-responded with *VviHDR* and *VviUGT85A2L4* (*VviGT14*) with correlation coefficients of over 0.78. The transcript for XM_002285337.1 (ERF003) was negatively correlated with the accumulation of four transcripts related to ABA biosynthesis/response and one transcript related to ethylene response (XM_002281384.2). Additionally, XM_002266159.1 (trihelix transcription factor GT-2) was negatively correlated with an ethylene-responsive transcription factor 1B (XM_002264487.1).

Researchers have previously reported that the accumulation of free and glycosidically bound monoterpenes is closely associated with grape maturity [2, 8, 15, 77]. Additionally, the concentration of terpenes is greatly affected by growing conditions and climate [17, 78, 79]. As observed in this study, the concentration of terpenoids varied between the years of 2010 and 2011, but both free and glycosidically bound terpene concentrations in the berries of the GT region were lower than those in the CL region over the two years. We thus infer that particular climate conditions (e.g., extreme drought) in the grape-growing season in the GT region accelerate the maturation process of grape berries through stimulating a series of ripening-related cues, such as the transcriptional activation of ripening-related genes, and the latter cascades regulatory factors and terpene biosynthesis-related genes and eventually limits the production of terpene volatiles.

### Quantitative real-time PCR

To validate the expression profiles obtained from RNA-seq, we performed qRT-PCR, on nine important genes associated with terpene biosynthesis, including *VviUGT85A2L4* (*VviGT14*), *VviUGT88A1L3* (*VviGT7)*, *VviGPPS*, *VviFPPS*, *VviPNLNGL1*, *VviCSLin/Ner*, *VviPLG1*,*VviNCED1* and *VviNCED2*. Three internal reference genes (*VviUbiquitin*, *VviActin* and *VviGADPH*) were applied. A good correlation was observed between the expression levels of these genes based on RPKM values and those determined by qRT-PCR (R^2^ > 0.7, Pearson correlation) (Additional file 2: Figure S2). This result demonstrated the reliablity of RNA-seq analysis.

## Conclusions

The present study demonstrated that both free and glycosidically bound terpene levels increased during the development of ‘Muscat Blanc a Petits Grains’ grapes. The genes which transcript accumulation patterns were consistent with the production of terpene volatiles were identified from the RNA-seq data, such as *VviHDR* and *VviUGT85A2L4* (*VviGT14*). The concentrations of terpenes, particularly in their glycosidically bound form, in the berries of CL region were significantly higher than in the GT region. The differential accumulation of glycosidically bound monoterpenes in the berries between the two regions and between the two years was closely related to the expression of *VviUGT85A2L4* (*VviGT14*), which encodes a monoterpenol glucosyltransferase. Putative TFs regulating the expression of *VviUGT85A2L4* (*VviGT14*) were identified through co-expression network analysis, and *Vvi*GT-2 L (XM_002266159.1, a trihelix TF) was found to highly correlate with the expression of *VviGT14*. At the initiation of veraison (E-L35), many genes required for the biosynthesis and signal transduction of ABA and ethylene were up-regulated at the transcriptional level in the berries of the GT region relative to the CL region. Based on the gene co-expression network analysis, a cascade process was constructed to interpret the mechanism underlying differential accumulation of terpenes between the berries grown in the two regions, which involved the effects of regional climate, the production of ripening-related hormones, the acceleration of berry ripening and the expression of terpene biosynthesis-associated genes and potential transcription factors. Although more evidences are required to validate this cascade link predicted herein, the present study proposed some key genes for differential terpene accumulation across two regions through the combined analysis of transcripts and metabolites. This work provides an entry point for further study about the regulation of terpene biosynthesis in muscat-type grape cultivars. These genes and transcription factors may prove useful as targets for grape aromatic improvement and/or biotechnology industry interests.

## Methods

### Sampling locations

‘Muscat Blanc à Petits Grains’ (*Vitis vinifera* L. Muscat blanc) is a white grape variety, and the mature berries are famous for their distinctive Muscat aroma. In the present study, grape berries were sampled from the vineyards located in the GT region (39°14′ N, 99°84′ E) of Gansu province and the CL region (39°72′N, 119°15′E) of Hebei province, China. The main geographical and climate information of these two regions is provided in Additional file 1: Table S8. In general, compared with the GT region, the CL region had a relatively higher average monthly and total effective accumulated temperature in the grape growth season. However, there exists significantly more sunshine hours and much less rainfall in the GT region.

### Grape materials

In either of the two regions, a vineyard with approximately 200 hectares was selected for this study. The vines in the studied vineyard were planted from cutting stems in 2001 (in GT) and 2006 (in CL), respectively. These grapevines were all trained on a vertical shoot positioning (VSP), arranged in north–south oriented rows spaced 2.0 m apart, with a distance of approximately 1.0 m between two plants in each row. The management of the vineyards was in accordance with the local wine grape cultivation practices. During the experimental period, similar disease and pest management as well as fertilization were carried out in the studied vineyards. Canopy manipulation was both performed manually according to vine growth. Each grapevine contained a main vine with 10–12 fruiting branches. All the field work got permission from the vineyard managers. Each vineyard was divided into two biological communities for grape sampling. In either of the two vineyards, the sampling was performed in the same vines in 2010 and 2011. Grape berries were collected at four time points: (1) pea-size berries (E-L stage 31), (2) berries beginning to color and enlarge (E-L stage 35), (3) berries with intermediate Brix values (E-L stage 36), and (4) ripe/harvest stage (E-L stage 38), respectively, with two repeats. The E-L stages were determined as described by Coombe [80]. To obtain a sample representing the vineyard population, approximately 1000 berries were randomly sampled from at least 200 vines in each plot at each stage. Any physically injured, abnormal or infected berries were excluded. Sampling time was at 10:00–11:00 in the morning. Samples were placed into a Ziplack bag and then put in the foam ice boxes, transported to experimental stations within two hours, rapidly frozen in liquid nitrogen and maintained at −80 °C. These samples were then transported back to the laboratory in the frozen state and all sampling was gathered by the end of each vintage, which totaled up to 32 samples consisting of two biological repeats at four developmental stages from two regions in two years.

### Physicochemical analysis

For each sample, approximately 50 g of berries with seed-removal in advance were homogenized in liquid nitrogen. The homogenate was used for the analyses of total soluble solids (TSS), titratable acidity (TA) and pH value. TSS was determined with an automatic temperature-compensated digital refractometer (Pocket Refractometer Pal-1, Atago, Japan), and the results were expressed as °Brix. TA and pH values were determined using a potentiometric titrator PB-10 (Sartorius, Germany). A sample of 5 mL clear juice was diluted with 50 mL de-ionized water and then used to determine titratable acidity. NaOH (0.05 mol/L) was added to an end-point titration of pH = 8.2, and the TA was calculated from the NaOH consumption volume. The content of TA was expressed as the equivalent of malic acid. Replicate measurements of each sample were performed.

### Extraction of free and glycosidically bound volatile compounds

Fifty frozen grape berries without seeds were smashed to powder in liquid nitrogen. After maceration for 120 min at 4 °C, the juice was centrifuged at 6000 *× g* for 10 min. Five mL of supernatant was blended with 1 g NaCl and 10 μL 4-methyl-2-pentanol (4M2P, 1.0018 g/L as an internal standard) in a 15-mL sample vial. The free volatiles of the prepared sample were extracted and concentrated using headspace SPME according to our previous study [81, 82]. Three independent extractions were performed for each sample.

The bound aromatic compounds were isolated through absorption on Cleanert PEP-SPE resins (Bonna-agela Technologies, China, 200 mg/6 mL) conditioned in advance with methanol and water (10 mL of each). Five milliliters of the clear juice was passed through the Cleanert PEP-SPE column. Water-soluble compounds were eluted with 5 mL of water, free volatiles with 10 mL of dichloromethane and aromatic precursors with 20 mL of methanol. The flow rate was approximately 2 mL/min. The methanol eluate was concentrated to dryness by an rotary evaporator under a vacuum and then re-dissolved in 5 mL of 2 mol/L citrate-phosphate buffer solution (pH 5.0). Subsequently, 100 μL of AR 2000 (Rapidase, DSM Food Specialties, France) solution (100 mg/mL in 2 mol/L citrate-phosphate buffer, pH 5.0) was added to the glycoside extract, and the mixture was vortexed. Enzymatic hydrolysis was performed under optimum conditions. The tube containing the mixture was sealed and placed in an incubator at 40 °C for 16 h to liberate free volatiles. The resultant free volatiles were extracted according to the SPME method mentioned above.

### GC-MS conditions

The volatile analysis was performed on an Agilent 7890 N gas chromatograph coupled to a 5975C mass spectrometer (Agilent Technologies, Santa Clara,Califonia, USA) and fitted with a 60 m × 0.25 mm id HP-INNOWAX capillary column with 0.25 μm film thickness (J&W Scientific, Folsom, CA, USA). The flow rate of the carrier gas (Helium) was 1 ml/min, and the SPME extracts were injected into the GC port at a splitless mode. The operating conditions were as follows: injector, 250 °C; ion source, 230 °C; interface, 280 °C. The temperature program was from 50 °C (1 min hold) to 220 °C at 3 °C /min and held at 220 °C for 5 min. Retention indices were calculated after analyzing the C6-C24 n-alkane series (Supelco, Bellefonte, PA, USA) under the same chromatographic conditions. Identifications were based on mass spectra matching in the standard NIST05 library and retention indices of reference standards in the authors’ laboratories. When reference standards were not available, tentative identifications were performed based on the standard NIST05 library and a comparison to retention indices reported in the literature (Additional file 1: Table S9).

### RNA library construction and sequencing

Approximately 50 berries were randomly selected from a 1000-berry biological replicate for RNA extraction. Total RNA was isolated from frozen grape berries without seeds using a plant RNA isolation kit (Sigma RT-250, St. Louis, MO, USA). RNA integrity was verified by agarose gel electrophoresis. RNA quantity and quality were assessed using a Qubit 2.0 fluorometer RNA Assay Kit (Invitrogen Inc. USA) and an Agilent 2100 Bioanalyzer RNA 6000 Nano kit (Agilent, USA). The Gene Expression Sample Prep Kit (IlluminaInc; San Diego, CA, USA) was used for sequence tag preparation according to the manufacturer's protocol, which is also well described by Zhong et al. [83]. Strand-specific RNA-seq libraries of approximately 200 bp fragments were constructed using 10 μg total RNA following the Cold Spring Harbor Protocols [83].

A total of 24 RNA-seq libraries were constructed and used for RNA-seq analysis in this study, consisting of four libraries corresponding to the grapes of E-L 31stage from GT and CL regions in the two vintages, eight for the E-L35 grapes, four for the E-L 36 grapes and eight for E-L 38 grapes. That is, with regard to the grapes at either E-L31 or E-L36 stage, only one RNA-seq library was obtained respectively for each region each year because of the small amount of high quality RNA acquired, while two libraries were acquired for the grapes at either E-L35 or E-L38 stage. Equal quantities of dsDNA from each library with different set of indexed primers were combined into two separate pools. Sequencing was performed on an Illumina HiSeq2000 instrument at the Cornell University Life Sciences Core Laboratories Center (USA). The sequencing data was deposited in the NCBI Sequence Read Archive (SRA) sequence database with accession number SRP061365.

### Mapping of Illumina sequence reads

Clean reads were mapped onto the reference sequence nucleotide collection (*Vitis vinifera* RefSeq mRNAs, consisting of 23,720 annotated transcripts) retrieved from the National Centre for Biotechnology Information (http://ncbi.nlm.nih.gov) for annotation using a CLC genomic workbench (CLC bio, Boston, USA). Considering the incomplete annotation of TPSs in the *Vitis vinifera* RefSeq database, the mRNA sequences of TPSs were downloaded from the grape genome database (V1) hosted at CRIBI (http://genomes.cribi.unipd.it/grape/), which consisted of 106 annotated transcripts that comprised the second reference dataset for our mapping.

Prior to transcriptome mapping, two nucleotides were trimmed from both ends of each sequence read. The reads under 60 nucleotides in length or with greater than two ambiguous nucleotides were excluded in mapping or counting. In this experiment, we run the assembly with the default mapping parameters allowing for a maximum of two mismatches and the maximum of ten hits for a read. Gene expression levels were represented by RPKM (reads per exon kilo base per million mapped sequence reads) values [84]. When reads could be mapped to multiple reference locations, they were assigned to reference transcripts proportionally based on the relative number of unique reads previously mapped to each of the reference sequences.

### Differential expression analysis of genes

Gene expression levels in developing grape berries were normalized and calculated as clean reads per kb per million reads (RPKM) values during the assembly and clustering processes. The data have been deposited in the NCBI Gene ExpressionOmnibus (GEO) database and are accessible through GEO accession GSE71146. P-values were used to evaluate the authenticity of differential transcript abundance. Bonferroni-corrected p-values were applied to control the false discovery rate (FDR) in multiple testing. “FDR ≤ 0.05 and absolute value log2-Ratio ≥ 1” was set as the threshold to judge the significance of gene expression difference between two samples. The default value (read number) of genes that were not identified in one of the samples was one.

### cDNA synthesis and quantitative real-time PCR analysis

Five micrograms of total RNA was used to synthesize first strand cDNA using the SuperScript first-strand synthesis system for quantitative real-time PCR (qRT-PCR) (Promaga, Madison, Wisconsin, USA). Two microliters of cDNA (100 ng/μL) were used for qRT-PCR using the SYBR Green PCR master mix (Takara, Dalian, China) following the manufacturer’s protocol and an ABI Real-time 7300 system (Applied Biosystems). qRT-PCR was performed on two independent biological replicates, each containing three technical replicates. Gene-specific oligonucleotide primers were designed using the PerPrimer version 2.0 software. Primer information is available in Additional file 1: Table S10. Three grapevine reference genes coding for GAPDH (EC930334), actin (EC969944) and ubiquitin (EC929411) were applied. A final volume of 20 μL PCR solution was composed of 10 μL of SYBR®Premix Ex TaqTM and 0.5 μL of ROX Reference Dye (50×) (Takara, Dalian, China), 1 μL of primer mixture (forward primer and reverse primer, 10 mM), 4 μL of diluted template cDNA and 4.5 μLddH2O. The PCR cycling conditions were: an initial denaturation step at 95 °C for 30 s, followed by 40 cycles of amplification at 94 °C for 10 s, followed by 60 °C for 31 s, and melt curve analysis from 65 °C to 95 °C to detect possible primer dimers or nonspecific amplification in cDNA samples. The specificity of the primers was verified by agarose gel electrophoresis and sequencing the reaction products. The expression level of target genes were calculated using the formula 2-∆CT, in which ∆CT = CT,target –CT,ref. and CT,ref was the geometric mean of three reference gene threshold cycles (CTs). The means and standard derivations (SD) were estimated after 2-∆CT calculations.

### Data analysis tools

The R software (version 2.0) was used for hierarchical cluster analysis, heatmap visualization, K means clustering and Pearson correlation evaluation. Co-expression networks were visualized with the Cytoscape software [85], v2.8.2 (www.cytoscape.org). A one-way analysis of variance (ANOVA) was used to measure differences between means of volatile concentrations employing Duncan’s multiple range tests at a level of *p* < 0.05. Data are presented as the means ± SDs (standard deviations). The phylogenetic tree was constructed by the neighbor-joining method with MEGA5.0 (molecular evolutionary genetics analysis).

## Availability of supporting data

The data sets supporting the metabolome results of this article are included within the article and its additional files. The RNA sequence data were downloaded from Gene Expression Omnibus (GEO) using accession number GSE71146 at website http://www.ncbi.nlm.nih.gov/geo/query/acc.cgi?acc=GSE71146.

## Additional files

Additional file 1: Table S1A. Statistical analysis of free terpene in ‘Muscat Blanc a Petits Grains’ berries in vitage 2010 and 2011. **Table S1B.** Statistical analysis of glycosidically-bound terpene in ‘Muscat Blanc a Petits Grains’ berries. **Table S1C.** Odour activity valuesa (OAVs) of most potent terpene volatiles in ripenning‘Muscat Blanc a Petits Grains’berries. **Table S2.** List of differentially expressed terpene metabolism related genes in 'Muscat Blanc a Petits Grains’ berries between CL and GT regions(GT/CL). **Table S3.** The Pearson's correlation coefficients between glucosyltransferase gene expression profiles and monoterpenes concentration. **Table S4A.** The information of UGT genes selected by phylogeny tree that showed high homology with the monoterpene glutransferease. **Table S4B.** The information of UGT genes selected by K means analysis. **Table S5.** Pearson correlation of transcriptional factors and selected genes (*p* < 0.05). **Table S6.** Differentailly-expressed transcript factor genes for the two regions at various developmental stages of grapes and their correlation with the expression of some stuctural genes in the terpene biosynthetic pathway. **Table S7.** Differentially-expressed genes in ABA/ethylene biosynthesis and signalling transduction pathway and their expression fold-changes. **Table S8.** Geographical location, soil type and climate condition of the two wine-growing regions. **Table S9.** List of Authentic standards and retention index run in GC-MS machine. **Table S10.** GenBank accession number and primers of amplified DNA fragments of genes for quantitative real-time PCR (qPCR). (XLSX 101 kb) Additional file 2: Figure S1. Predication of putative monoterpenol glucosyltransferase. (A) k-means cluster of the UDP-glycosyltransferase (UGTs) transcripts in ‘Muscat Blanc a Petits Grains’. (B) phylogeny tree of UGTs based on amino acid sequences. Protein sequences are from *vitis vinifera* with known glucosyltransferase activity toward terpenes and biochemically characterized proteins from Vitis spp. (*Vitis vinifera* [Vvi] and *Vitis labrusca* [Vl]). **Figure S2.** The genes showed high homology with known terpene GTs were marked with color. Correlation of gene expression reported by the RNA-Seq and by quantitative Real-Time PCR. Data were from nine genes across four developmental stages in two years. Both the RNA-Seq values and the qRTPCR values were normalized with log2, and linear regression analysis gave an overall coefficient of variation of each gene. (ZIP 735 kb)

## Abbreviations

GT: Gaotai region of Gansu province, China
CL: Changli region of Hebei province, China
MEP: 2-methyl-D-erythritol-4-phosphate phosphate
MVA: Mevalonic acid
DXS: 1-Deoxy-D-xylulose 5-phosphate synthase
HDR: 1-hydroxy-2-methyl-2-butenyl 4-diphosphate reductase
TPS: Terpene synthase
GT: monoterpene glucosyltransferase
TF: Transcription factor
DEGs: Differentially-expressed genes
GC-MS: Gas chromatography coupled to mass spectrometry

## References

[1] Strauss CR, Wilson B, Gooley PR, Williams PJ (1986). Role of monoterpenes in grape and wine flavor.

[2] Robinson AL, Boss PK, Solomon PS, Trengove RD, Heymann H, Ebeler SE (2014). Origins of grape and wine aroma. Part 1. chemical components and viticultural impacts. Am J Enol Viticult.

[3] Dimitriadis E, Williams P (1984). The development and use of a rapid analytical technique for estimation of free and potentially volatile monoterpene flavorants of grapes. Am J Enol Viticult.

[4] Gunata Y, Bayonove C, Baumes R, Cordonnier R (1985). The aroma of grapes I. Extraction and determination of free and glycosidically bound fractions of some grape aroma components. J Chromatogr A.

[5] Gunata Y, Bayonove C, Baumes R, Cordonnier R (1986). Stability of free and bound fractions of some aroma components of grapes cv. Muscat during the wine processing: preliminary results. Am J Enol Viticult.

[6] Maicas S, Mateo JJ (2005). Hydrolysis of terpenyl glycosides in grape juice and other fruit juices: a review. Appl Microbiol Biot.

[7] Ribéreau-Gayon P, Boidron J, Terrier A (1975). Aroma of Muscat grape varieties. J Agr Food Chem.

[8] Fenoll J, Manso A, Hellín P, Ruiz L, Flores P (2009). Changes in the aromatic composition of the *Vitis vinifera* grape Muscat Hamburg during ripening. Food Chem.

[9] Palomo ES, Pérez-Coello M, Díaz-Maroto M, González Viñas M, Cabezudo M (2006). Contribution of free and glycosidically-bound volatile compounds to the aroma of muscat “a petit grains” wines and effect of skin contact. Food Chem.

[10] Voirin SG, Baumes RL, Sapis JC, Bayonove CL (1992). Analytical methods for monoterpene glycosides in grape and wine 1: II. Qualitative and quantitative determination of monoterpene glycosides in grape. J Chromatogr A.

[11] Martin DM, Chiang A, Lund ST, Bohlmann J (2012). Biosynthesis of wine aroma: transcript profiles of hydroxymethylbutenyl diphosphate reductase, geranyl diphosphate synthase, and linalool/nerolidol synthase parallel monoterpenol glycoside accumulation in Gewurztraminer grapes. Planta.

[12] Skinkis PA, Bordelon BP, Butz EM (2010). Effects of sunlight exposure on berry and wine monoterpenes and sensory characteristics of Traminette. Am J Enol Viticult.

[13] Zhang H, Fan P, Liu C, Wu B, Li S, Liang Z (2014). Sunlight exclusion from Muscat grape alters volatile profiles during berry development. Food Chem..

[14] García-Muñoz S, Asproudi A, Cabello F, Borsa D (2011). Aromatic characterization and enological potential of 21 minor varieties (*Vitis vinifera* L.). Eur Food Res Technol.

[15] Robinson A (2011). Environmental influences on grape aroma potential.

[16] Vilanova M, Genisheva Z, Bescansa L, Masa A, Oliveira JM (2012). Changes in free and bound fractions of aroma compounds of four *Vitis vinifera* cultivars at the last ripening stages. Phytochemistry.

[17] Reynolds AG, Wardle DA (1989). Impact of various canopy manipulation techniques on growth, yield, fruit composition, and wine quality of Gewürztraminer. Am J Enol Viticult.

[18] Deluc LG, Quilici DR, Decendit A, Grimplet J, Wheatley MD, Schlauch KA (2009). Water deficit alters differentially metabolic pathways affecting important flavor and quality traits in grape berries of Cabernet Sauvignon and Chardonnay. BMC Genomics.

[19] Xu X-Q, Liu B, Zhu B-Q, Lan Y-B, Gao Y, Wang D (2015). Differences in volatile profiles of Cabernet Sauvignon grapes grown in two distinct regions of China and their responses to weather conditions. Plant Physiol Biochem.

[20] Jiang B, Xi Z, Luo M, Zhang Z (2013). Comparison on aroma compounds in Cabernet Sauvignon and Merlot wines from four wine grape-growing regions in China. Food Res Int.

[21] McGarvey DJ, Croteau R (1995). Terpenoid metabolism. Plant Cell.

[22] Lichtenthaler HK (1999). The 1-deoxy-D-xylulose-5-phosphate pathway of isoprenoid biosynthesis in plants. Annu Rev Plant Biol.

[23] Muhlemann JK, Klempien A, Dudareva N (2014). Floral volatiles: from biosynthesis to function. Plant Cell Environ.

[24] Bohlmann J, Keeling CI (2008). Terpenoid biomaterials. Plant J.

[25] Luan F, Wüst M (2002). Differential incorporation of 1-deoxy-d-xylulose into (3S)-linalool and geraniol in grape berry exocarp and mesocarp. Phytochemistry.

[26] Vranová E, Coman D, Gruissem W (2013). Network analysis of the MVA and MEP pathways for isoprenoid synthesis. Annu Rev Plant Biol.

[27] Vranová E, Coman D, Gruissem W (2012). Structure and dynamics of the isoprenoid pathway network. Mol Plant.

[28] Mateo J, Jiménez M (2000). Monoterpenes in grape juice and wines. J Chromatogr A.

[29] Rapp A (1998). Volatile flavour of wine: correlation between instrumental analysis and sensory perception. Nahrung.

[30] Botella‐Pavía P, Besumbes O, Phillips MA, Carretero‐Paulet L, Boronat A, Rodríguez‐Concepción M (2004). Regulation of carotenoid biosynthesis in plants: evidence for a key role of hydroxymethylbutenyl diphosphate reductase in controlling the supply of plastidial isoprenoid precursors. Plant J.

[31] Estévez JM, Cantero A, Reindl A, Reichler S, León P (2001). 1-Deoxy-D-xylulose-5-phosphate synthase, a limiting enzyme for plastidic isoprenoid biosynthesis in plants. J Biol Chem.

[32] Mahmoud SS, Croteau RB (2001). Metabolic engineering of essential oil yield and composition in mint by altering expression of deoxyxylulose phosphate reductoisomerase and menthofuran synthase. Proc Natl Acad Sci.

[33] Wright L, Rohwer JM, Ghirardo A, Hammerbacher A, Ortíz M, Raguschke B (2014). 1-Deoxyxylulose 5-phosphate synthase controls flux through the 2-C-methylerythritol 4-phosphate pathway in *Arabidopsis thaliana*. Plant Physiol..

[34] Battilana J, Emanuelli F, Gambino G, Gribaudo I, Gasperi F, Boss PK (2011). Functional effect of grapevine 1-deoxy-D-xylulose 5-phosphate synthase substitution K284N on Muscat flavour formation. J Exp Bot.

[35] Battilana J, Costantini L, Emanuelli F, Sevini F, Segala C, Moser S (2009). The 1-deoxy-d-xylulose 5-phosphate synthase gene co-localizes with a major QTL affecting monoterpene content in grapevine. Theor Appl Genet.

[36] Emanuelli F, Battilana J, Costantini L, Le Cunff L, Boursiquot J-M, This P (2010). A candidate gene association study on muscat flavor in grapevine (*Vitis vinifera* L.). BMC Plant Biol.

[37] Tholl D (2006). Terpene synthases and the regulation, diversity and biological roles of terpene metabolism. Curr Opin Plant Biol.

[38] Lange BM, Wildung MR, Stauber EJ, Sanchez C, Pouchnik D, Croteau R (2000). Probing essential oil biosynthesis and secretion by functional evaluation of expressed sequence tags from mint glandular trichomes. Proc Natl Acad Sci.

[39] Martin DM, Aubourg S, Schouwey MB, Daviet L, Schalk M, Toub O (2010). Functional annotation, genome organization and phylogeny of the grapevine (*Vitis vinifera*) terpene synthase gene family based on genome assembly, FLcDNA cloning, and enzyme assays. BMC Plant Biol.

[40] Lücker J, Bowen P, Bohlmann J (2004). *Vitis vinifera* terpenoid cyclases: functional identification of two sesquiterpene synthase cDNAs encoding (+)-valencene synthase and (−)-germacrene D synthase and expression of mono- and sesquiterpene synthases in grapevine flowers and berries. Phytochemistry.

[41] Martin DM, Bohlmann J (2004). Identification of *Vitis vinifera* (−)-alpha-terpineol synthase by in silico screening of full-length cDNA ESTs and functional characterization of recombinant terpene synthase. Phytochemistry.

[42] Matarese F, Scalabrelli G, D’Onofrio C (2013). Analysis of the expression of terpene synthase genes in relation to aroma content in two aromatic *Vitis vinifera* varieties. Funct Plant Biol.

[43] Bönisch F, Frotscher J, Stanitzek S, Rühl E, Wüst M, Bitz O (2014). A UDP-Glucose:monoterpenol glucosyltransferase adds to the chemical diversity of the grapevine metabolome. Plant Physiol.

[44] Bönisch F, Frotscher J, Stanitzek S, Rühl E, Wüst M, Bitz O (2014). Activity based profiling of a physiologic aglycone library reveals sugar acceptor promiscuity of family 1 UDP-glucosyltransferases from *Vitis vinifera*. Plant Physiol.

[45] Li Z, Pan Q, Jin Z, Mu L, Duan C (2011). Comparison on phenolic compounds in *Vitis vinifera* cv. Cabernet Sauvignon wines from five wine-growing regions in China. Food Chem.

[46] Sun R, He F, Lan Y, Xing R, Liu R, Pan Q (2015). Transcriptome comparison of Cabernet Sauvignon grape berries from two regions with distinct climate. J Plant Physiol..

[47] Wilson B, Strauss CR, Williams P (1986). The distribution of free and glycosidically-bound monoterpenes among skin, juice, and pulp fractions of some white grape varieties. Am J Enol Viticult.

[48] Williams P, Strauss C, Wilson B (1981). Classification of the monoterpenoid composition of Muscat grapes. Am J Enol Viticult.

[49] Wilson B, Strauss CR, Williams PJ (1984). Changes in free and glycosidically bound monoterpenes in developing Muscat grapes. J Agr Food Chem.

[50] Rohmer M (1999). The discovery of a mevalonate-independent pathway for isoprenoid biosynthesis in bacteria, algae and higher plants. Nat Prod Rep.

[51] Ro D-K, Paradise EM, Ouellet M, Fisher KJ, Newman KL, Ndungu JM (2006). Production of the antimalarial drug precursor artemisinic acid in engineered yeast. Nature.

[52] Sweetman C, Wong DCJ, Ford CM, Drew DP (2012). Transcriptome analysis at four developmental stages of grape berry (*Vitis vinifera* cv. Shiraz) provides insights into regulated and coordinated gene expression. Bmc Genomics.

[53] Guillaumie S, Fouquet R, Kappel C, Camps C, Terrier N, Moncomble D (2011). Transcriptional analysis of late ripening stages of grapevine berry. BMC Plant Biol.

[54] Falara V, Akhtar TA, Nguyen TT, Spyropoulou EA, Bleeker PM, Schauvinhold I (2011). The tomato terpene synthase gene family. Plant Physiol.

[55] Matarese F, Cuzzola A, Scalabrelli G, D'Onofrio C (2014). Expression of terpene synthase genes associated with the formation of volatiles in different organs of *Vitis vinifera*. Phytochemistry..

[56] Zhu B-Q, Cai J, Wang Z-Q, Xu X-Q, Duan C-Q, Pan Q-H (2014). Identification of a plastid-localized bifunctional nerolidol/linalool synthase in relation to linalool biosynthesis in young grape berries. Int J Mol Sci.

[57] Grimplet J, Adam-Blondon A-F, Bert P-F, Bitz O, Cantu D, Davies C (2014). The grapevine nomenclature system. BMC Genomics..

[58] Xu YH, Wang JW, Wang S, Wang JY, Chen XY (2004). Characterization of GaWRKY1, a cotton transcription factor that regulates the sesquiterpene synthase gene (+)-delta-cadinene synthase-A. Plant Physiol.

[59] Spyropoulou EA, Haring MA, Schuurink RC (2014). RNA sequencing on *Solanum lycopersicum* trichomes identifies transcription factors that activate terpene synthase promoters. BMC Genomics.

[60] Yu Z-X, Li J-X, Yang C-Q, Hu W-L, Wang L-J, Chen X-Y (2012). The jasmonate-responsive AP2/ERF transcription factors AaERF1 and AaERF2 positively regulate artemisinin biosynthesis in *artemisia annua* L. Molecular Plant.

[61] Reeves PH, Ellis CM, Ploense SE, Wu M-F, Yadav V, Tholl D (2012). A regulatory network for coordinated flower maturation. PLoS Genet.

[62] Hong G-J, Xue X-Y, Mao Y-B, Wang L-J, Chen X-Y (2012). Arabidopsis MYC2 interacts with DELLA proteins in regulating sesquiterpene synthase gene expression. Plant Cell.

[63] Hichri I, Heppel SC, Pillet J, Leon C, Czemmel S, Delrot S (2010). The basic Helix-Loop-Helix Ttranscription factor MYC1 is involved in the regulation of the flavonoid biosynthesis pathway in grapevine. Mol Plant.

[64] Zhang H, Jin J, Tang L, Zhao Y, Gu X, Gao G (2011). PlantTFDB 2.0: update and improvement of the comprehensive plant transcription factor database. Nucleic Acids Res.

[65] Kaplan-Levy RN, Brewer PB, Quon T, Smyth DR (2012). The trihelix family of transcription factors – light, stress and development. Trends Plant Sci.

[66] Galon Y, Aloni R, Nachmias D, Snir O, Feldmesser E, Scrase-Field S (2010). Calmodulin-binding transcription activator 1 mediates auxin signaling and responds to stresses in Arabidopsis. Planta.

[67] Yang T, Poovaiah B (2002). A calmodulin-binding/CGCG box DNA-binding protein family involved in multiple signaling pathways in plants. J Biol Chem.

[68] Carbonell-Bejerano P, Santa María E, Torres-Pérez R, Royo C, Lijavetzky D, Bravo G (2013). Thermotolerance responses in ripening berries of *Vitis vinifera* L. cv Muscat Hamburg. Plant Cell Physiol.

[69] Mannen K, Matsumoto T, Takahashi S, Yamaguchi Y, Tsukagoshi M, Sano R (2014). Coordinated transcriptional regulation of isopentenyl diphosphate biosynthetic pathway enzymes in plastids by phytochrome-interacting factor 5. Biochem Biophys Res Commun.

[70] Valdés A, Övernäs E, Johansson H, Rada-Iglesias A, Engström P (2012). The homeodomain-leucine zipper (HD-Zip) class I transcription factors ATHB7 and ATHB12 modulate abscisic acid signalling by regulating protein phosphatase 2C and abscisic acid receptor gene activities. Plant Mol Biol.

[71] Zahur M, Asif MA, Zeeshan N, Mehmood S, Malik MF, Asif AR (2013). Homeobox leucine zipper proteins and cotton improvement. Adv Biosci Biotechnol.

[72] Young PR, Lashbrooke JG, Alexandersson E, Jacobson D, Moser C, Velasco R (2012). The genes and enzymes of the carotenoid metabolic pathway in *Vitis vinifera* L. BMC Genomics.

[73] Kuhn N, Guan L, Dai ZW, Wu B-H, Lauvergeat V, Gomès E (2014). Berry ripening: recently heard through the grapevine. J Exp Bot.

[74] Chervin C, El-Kereamy A, Roustan J-P, Latché A, Lamon J, Bouzayen M (2004). Ethylene seems required for the berry development and ripening in grape, a non-climacteric fruit. Plant Sci.

[75] Sun L, Zhang M, Ren J, Qi J, Zhang G, Leng P (2010). Reciprocity between abscisic acid and ethylene at the onset of berry ripening and after harvest. BMC Plant Biol.

[76] Yamane T, Jeong ST, Goto-Yamamoto N, Koshita Y, Kobayashi S (2006). Effects of temperature on anthocyanin biosynthesis in grape berry skins. Am J Enol Viticult.

[77] Fenoll J, Maria Martinez C, Hellin P, Flores P (2012). Changes of free and glycosidically bound monoterpenes and aromatic alcohols in Moscatuel and Ruby seedless table grapes during development. J Int Sci Vigne Vin.

[78] Reynolds AG, Wardle DA (1989). Influence of fruit microclimate on monoterpene levels of Gewürztraminer. Am J Enol Viticult.

[79] Gerdes SM, Winterhalter P, Ebeler SE (2002). Effect of sunlight exposure on norisoprenoid formation in White Riesling grapes.

[80] Coombe B (1995). Growth stages of the grapevine: adoption of a system for identifying grapevine growth stages. Aust J Grape Wine R.

[81] Wen Y-Q, He F, Zhu B-Q, Lan Y-B, Pan Q-H, Li C-Y (2014). Free and glycosidically bound aroma compounds in cherry (*Prunus avium* L.). Food Chem.

[82] Liu B, Xu X-Q, Cai J, Lan Y-B, Zhu B-Q, Wang J (2015). The free and enzyme-released volatile compounds of distinctive *Vitis amurensis* var. Zuoshanyi grapes in China. Eur Food Res Technol.

[83] Zhong S, Joung J-G, Zheng Y, Chen Y-r, Liu B, Shao Y (2011). High-throughput illumina strand-specific RNA sequencing library preparation. Cold Spring Harb Protoc.

[84] Mortazavi A, Williams BA, McCue K, Schaeffer L, Wold B (2008). Mapping and quantifying mammalian transcriptomes by RNA-Seq. Nat Methods.

[85] Shannon P, Markiel A, Ozier O, Baliga NS, Wang JT, Ramage D (2003). Cytoscape: a software environment for integrated models of biomolecular interaction networks. Genome Res.

