# Supplementary figures and images for: Using the combined analysis of transcripts and metabolites to propose key genes for differential terpene accumulation across two regions
Source: BMC Plant Biol. 2015 Oct 6;15:240. doi: 10.1186/s12870-015-0631-1 (PMC4595271; doi:10.1186/s12870-015-0631-1)

A


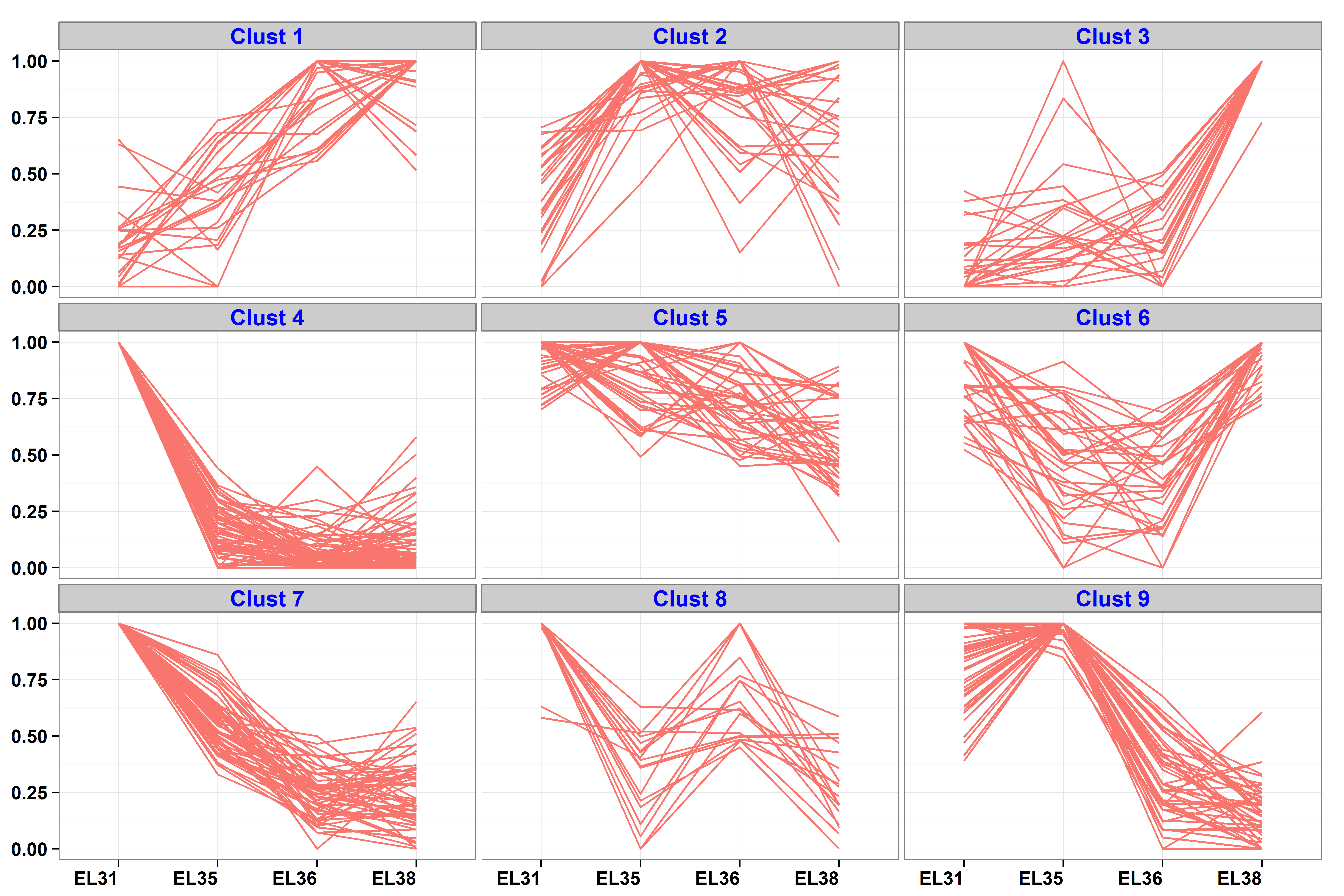


B

Additional file 2: Figure S1

Supplement: Additional file 2: Figure S1. — Predication of putative monoterpenol glucosyltransferase. (A) k-means cluster of the UDP-glycosyltransferase (UGTs) transcripts in ‘Muscat Blanc a Petits Grains’. (B) phylogeny tree of UGTs based on amino acid sequences. Protein sequences are from vitis vinifera with known glucosyltransferase activity toward terpenes and biochemically characterized proteins from Vitis spp. (Vitis vinifera [Vvi] and Vitis labrusca [Vl]). Figure S2. The genes showed high homology with known terpene GTs were marked with color. Correlation of gene expression reported by the RNA-Seq and by quantitative Real-Time PCR. Data were from nine genes across four developmental stages in two years. Both the RNA-Seq values and the qRTPCR values were normalized with log2, and linear regression analysis gave an overall coefficient of variation of each gene. (ZIP 735 kb) [file 12870_2015_631_MOESM2_ESM.zip › Additional file 2/Additional file 2 Figure S1 .docx]
